# Underreporting of blood and body fluid exposures among nurses: prevalence, reporting cascade, and modifiable organizational correlates

**DOI:** 10.3389/fpubh.2026.1989101

**Published:** 2026-10-07

**Authors:** Chunju Tang, Mei Zhang, Li Sun, Haiqin Pu, Xin Zhang, Yaqin Zhang, Hongyu Zhu

**Affiliations:** 1 Jiangsu College of Nursing, Huai'an, Jiangsu, China; 2 Department of Infection Control, Huai’an Fourth People’s Hospital, Huai'an, Jiangsu, China; 3 Department of Infection Control, The Fourth People’s Hospital of Lianyungang, The Affiliated KangDa College of Nanjing Medical University, Lianyungang, Jiangsu, China

**Keywords:** China, needlestick injuries, nurses, occupational exposure, patient safety culture, post-exposure prophylaxis, safety climate, underreporting

## Abstract

**Background:**

Occupational exposure to blood and body fluids is common among nurses, and incomplete reporting limits occupational surveillance and access to post-exposure assessment. This study estimated underreporting at the nurse level and the proportion of aggregate exposure counts that were unreported, and examined associated individual and workplace characteristics.

**Methods:**

A census-based analytical cross-sectional survey was conducted between November 2025 and March 2026 among registered nurses at a tertiary infectious disease referral hospital in China, using a structured questionnaire with a 12-month recall period. Underreporting was defined among exposed nurses as failure to formally report at least one exposure. Modified Poisson regression with robust variance was used to estimate adjusted prevalence ratios (aPRs). Negative-binomial and hurdle models with an exposure offset served as sensitivity analyses for exposure opportunity.

**Results:**

Of 1,110 eligible nurses, 872 responded (78.6%); 392 (45.0%) reported at least one exposure, contributing 747 aggregate exposure counts. Underreporting affected 232 exposed nurses (59.2, 95% CI 54.3–63.9), and 373 of 747 exposure counts (49.9%) were unreported. Among under-reporters, 48.3% reported receiving none of the five assessed services after any exposure during the recall period, compared with 0.6% of complete reporters; clinical indications and exposure-specific service receipt were unavailable. Underreporting was associated with individual perceptions of safety climate (aPR per SD 0.77, 95% CI 0.70–0.84), vague or absent procedural knowledge (aPR 1.56, 95% CI 1.31–1.86), non-permanent employment (aPR 1.30, 95% CI 1.07–1.57), and night-shift frequency (aPR per level 1.14, 95% CI 1.04–1.25).

**Conclusion:**

Underreporting was common at both the nurse level and across aggregate exposure counts. Perceived safety climate, procedural knowledge, employment security, and night-shift work were associated with underreporting and are potential targets for intervention research; the cross-sectional design does not establish causation or event-specific clinical consequences.

## 1 Introduction

Occupational exposure to blood and body fluids remains a substantial and potentially preventable hazard among healthcare workers, particularly nurses, whose frequent involvement in invasive procedures increases their susceptibility to percutaneous and mucocutaneous injuries. A meta-analysis encompassing 148 studies across 43 countries estimated the annual prevalence of percutaneous injury at 36.4% among healthcare workers, 43.2% in Asia, and 40.9% among nurses (1). Following percutaneous exposure to an infected source, transmission risk is approximately 0.3% for human immunodeficiency virus (HIV) and 6–40% for hepatitis B virus, depending on source antigen status (2). Globally, contaminated sharps injuries were estimated to account for 66,000 hepatitis B, 16,000 hepatitis C, and 1,000 HIV infections among healthcare workers in 2000, corresponding to attributable fractions of 37, 39, and 4.4%, respectively (3).

Timely institutional notification represents a critical component of occupational exposure management, facilitating risk assessment, baseline serological testing, hepatitis B immunoprophylaxis, HIV post-exposure prophylaxis, and subsequent clinical surveillance (4, 5). When an exposure remains unreported, opportunities for timely prophylaxis and appropriate follow-up may be missed, particularly when the infectious status of the source patient is positive or unknown. Furthermore, incomplete reporting distorts institutional surveillance, underestimates occupational risk, and compromises evidence-informed decisions regarding staffing, protective equipment, safety-engineered devices, and preventive training (46, 47). Despite these consequences, a meta-analysis of 41 studies involving 19,635 healthcare workers estimated that 59.9% of needlestick injuries remained unreported (95% CI, 52.0–67.7%) (6).

Evidence from China indicates substantial heterogeneity in occupational exposure and reporting practices. A national survey of 20,791 registered nurses found that 52.1% had experienced blood or body fluid exposure and that 34.6% of exposures were unreported; longer working hours and limited availability of protective equipment and safety-engineered devices were associated with greater exposure (7). A provincial investigation involving 6,464 healthcare workers reported an injury incidence of 32.9% and an underreporting prevalence of 28.5% (8). Conversely, a study of 223,149 workers across 360 hospitals documented institutional notification for only 4.6% of injuries (9), whereas a survey of 548 nurses in Changsha found that only 14.6% reported every exposure (10). Hollow-bore needles remain a predominant source of occupational sharps injuries, further underscoring the clinical importance of comprehensive exposure surveillance (11). Nevertheless, differences in study populations, reporting definitions, and analytical denominators complicate direct comparisons across these investigations.

Several methodological limitations further constrain interpretation of the available evidence. Reporting behavior is frequently reduced to a binary measure, obscuring distinctions between complete, partial, and absent reporting among nurses with repeated exposures. Moreover, underreporting is generally assessed only as an individual characteristic rather than as a proportion of all exposures experienced, preventing estimation of the share of exposures omitted from institutional surveillance. Few investigations examine whether reporting behavior corresponds to receipt of post-exposure assessment, prophylaxis, or follow-up services. Organizational determinants are also commonly represented by isolated indicators rather than multidimensional assessments of safety climate, psychological safety, and perceived institutional support, despite evidence that reporting culture and preventive infrastructure are relevant to occupational safety practice (12–14).

Dedicated infectious disease hospitals warrant consideration because their patient populations include individuals with HIV, hepatitis B, and hepatitis C, potentially increasing the clinical implications of unreported exposure (15). However, evidence integrating exposure burden, reporting behavior, post-exposure management, and organizational conditions within these settings remains limited. Accordingly, this single-center cross-sectional study evaluated nurses at a tertiary infectious disease hospital in China to quantify underreporting at the nurse level and as a proportion of aggregate exposure counts, describe reporting and post-exposure management, and identify potentially modifiable correlates of underreporting.

## 2 Materials and methods

### 2.1 Study design and setting

This single-center analytical cross-sectional study was conducted among registered nurses employed at a tertiary infectious disease referral hospital in China. It was reported in accordance with the Strengthening the Reporting of Observational Studies in Epidemiology statement for cross-sectional studies (16). Data were collected between November 2025 and March 2026. Verification against the original Mandarin questionnaire, its online branching logic, and the prespecified data dictionary confirmed that all exposure count and reporting questions referred to the 12 months preceding each respondent’s survey completion date. The questionnaire wording and corresponding variable definitions are reproduced in Supplementary Table S1.

The Fourth People’s Hospital of Lianyungang, Jiangsu Province, is a regional referral center for hepatitis B, hepatitis C, tuberculosis, HIV, and high-consequence respiratory infections. Conducting the study within one institution ensured that participants were nominally subject to the same formal reporting policy, occupational health service, and post-exposure prophylaxis pathway. However, the study measured variation in individual perceptions within one hospital and was not designed to estimate hospital-level contextual effects or the causal effects of modifying institutional policy.

### 2.2 Participants and sampling

Eligible participants were registered nurses who provided direct patient care and had worked at the hospital for at least 12 months, thereby ensuring employment throughout the complete 12-month exposure recall period. Nurses who had been on extended leave for the entire recall period, worked exclusively in administrative or educational positions, or were unregistered students were ineligible.

A census approach was used, and all eligible nurses were invited to participate; consequently, no probability sample was drawn, and no *a priori* sample-size calculation was performed. Of 1,240 nurses in the institutional establishment, 1,110 met the eligibility criteria and were invited, of whom 872 returned complete questionnaires (response rate 78.6%). The remaining 238 eligible nurses did not provide complete responses. Only their aggregate number was available; individual-level demographic, occupational, exposure, reporting, and organizational-perception data were unavailable. Respondent–non-respondent comparisons, non-response weighting, and quantitative adjustment for non-response were therefore not possible.

Three nested analytic populations were defined before analysis. The full cohort (*n* = 872) was used to describe participant characteristics, immunization and training status, organizational perceptions, and the prevalence of blood or body fluid (BBF) exposure. The exposed cohort comprised nurses reporting at least one BBF exposure during the preceding 12 months (*n* = 392) and was used to estimate nurse-level underreporting, aggregate reported and unreported exposure counts, characteristics of the most recent exposure, post-exposure service receipt, and associations with reporting. With 232 under-reporters, this cohort had more than 20 outcome events per model parameter (17). Analyses of reasons for not reporting and proposed facilitators were restricted to nurses who had failed to report at least one exposure (*n* = 232).

### 2.3 Survey instrument

Data were collected using a structured, self-administered questionnaire containing 45 numbered questions that generated 89 recorded variables across seven sections. These sections assessed demographic and occupational characteristics (Q1–Q10); hepatitis B immunization, biosafety training, knowledge of the institutional reporting procedure, and knowledge of post-exposure prophylaxis (PEP) access (Q11–Q15); percutaneous and mucocutaneous exposure counts, characteristics of the most recent exposure, and seven proximal contributing factors (Q16–Q24); reporting behavior and post-exposure management (Q25–Q31); 12 reasons for not reporting, the single most important reason, and eight potential reporting facilitators (Q32–Q34); organizational perceptions (Q35–Q37); and workload, working while exhausted or unwell, PPE burden, reporting intention, perceived infection risk, confidence in anonymity, and an optional free-text response (Q38–Q45).

Psychological safety, safety climate, and perceived organizational support were assessed using multi-item Likert scales ranging from 1 (“strongly disagree”) to 5 (“strongly agree”). The psychological safety scale contained seven items, including three negatively worded items that were reverse-scored; the safety-climate scale contained six items; and the perceived organizational support scale contained four items. Each scale was calculated as the unweighted mean of its constituent items, with higher scores representing more favorable perceptions. Scale scores were standardized to a mean of 0 and an SD of 1 for regression analysis.

The organizational scales were adapted from Edmondson’s team psychological safety scale (18), the safety-culture dimensions of the Hospital Survey on Patient Safety Culture (19), and the Survey of Perceived Organizational Support (20). Two bilingual nurse researchers independently translated the items into Mandarin, after which a third bilingual translator, blinded to the source wording, performed back-translation. Discrepancies were reconciled by the research team. Content validity was reviewed by eight experts comprising three infection-prevention specialists, three senior nurse managers, and two occupational health physicians. The revised questionnaire was piloted among 30 nurses who were excluded from the analytic sample.

The questionnaire was administered through the Wenjuanxing online platform (wjx.cn) using conditional branching. Exposure-specific questions were presented only to nurses reporting an exposure, while reporting- and non-reporting-specific questions were displayed only when logically applicable. No direct personal identifiers were collected. A substantive response to the optional free-text question was provided by 105 of 872 respondents (12.0%); these responses were not analyzed formally.

### 2.4 Variables and operational definitions

Two complementary underreporting estimands were defined. The nurse-level estimand was the prevalence of exposed nurses who failed to submit a formal institutional report for at least one BBF exposure during the 12-month recall period. For each nurse, unreported exposures were calculated as the exact total experienced (Q26a) minus the exact number reported (Q26b); values ≥1 defined under-reporters, and 0 defined complete reporters. Because additional exposures increase the opportunity to meet this definition, this estimand represented the prevalence of nurses affected by any underreporting, not the probability that an individual exposure was unreported. The exposure-opportunity estimand was the proportion of exposures that remained unreported, summarized as the aggregate proportion of exposure counts unreported and examined in sensitivity analyses relating each nurse’s unreported exposures to total exposures.

Q26a and Q26b recorded exact, non-negative, non-top-coded integer counts. The aggregate total of 747 exposures was obtained by summing Q26a; reported and unreported totals were obtained by summing Q26b and the respondent-level differences between Q26a and Q26b, respectively. The “five or more” percutaneous and mucocutaneous categories in Table 1 were presentation categories derived from the original integer variables and were not used to reconstruct the total. The 747 counts therefore do not represent independently characterized exposure records. Count-based classifications were compared with Q25, which asked whether respondents had reported all, most, some, or none of their exposures. Because both measures were completed by the same respondent in the same questionnaire and concerned the same recalled behavior, concordance was treated as an internal consistency check rather than as independent or external validation.

**Table 1:** Characteristics of blood and body fluid exposures, overall and by reporting status.

Variable	Category	Overall, *n* (%)	Reported all, *n* (%)	Underreported, *n* (%)	*p*-value
Type and frequency of exposure, past 12 months (overall *n* = 392; reported all *n* = 160; underreported *n* = 232)					
Percutaneous injuries	No percutaneous injuries	105 (26.8)	55 (34.4)	50 (21.6)	—
	One injury	161 (41.1)	70 (43.8)	91 (39.2)	
	Two injuries	87 (22.2)	29 (18.1)	58 (25.0)	
	Three injuries	34 (8.7)	6 (3.8)	28 (12.1)	
	Four injuries	4 (1.0)	0 (0.0)	4 (1.7)	
	Five or more injuries	1 (0.3)	0 (0.0)	1 (0.4)	
Mucocutaneous exposures	No mucocutaneous exposures	194 (49.5)	87 (54.4)	107 (46.1)	—
	One exposure	128 (32.7)	58 (36.2)	70 (30.2)	
	Two exposures	54 (13.8)	12 (7.5)	42 (18.1)	
	Three exposures	13 (3.3)	3 (1.9)	10 (4.3)	
	Four exposures	1 (0.3)	0 (0.0)	1 (0.4)	
	Five or more exposures	2 (0.5)	0 (0.0)	2 (0.9)	
Total exposure counts	Sum across respondents	747	237	510	—
Exposure per nurse	Mean (SD)	1.9 (1.1)	1.5 (0.7)	2.2 (1.2)	—
	Median [IQR]	2.0 [1.0–2.0]	1.0 [1.0–2.0]	2.0 [1.0–3.0]	—
Most recent percutaneous injury (overall *n* = 287; reported all *n* = 105; underreported *n* = 182)					
Device involved	Hollow-bore hypodermic needle	69 (24.0)	20 (19.0)	49 (26.9)	0.311
	Intravenous cannula or stylet	55 (19.2)	21 (20.0)	34 (18.7)	
	Blood collection needle or vacutainer	53 (18.5)	18 (17.1)	35 (19.2)	
	Lancet	31 (10.8)	11 (10.5)	20 (11.0)	
	Other	22 (7.7)	13 (12.4)	9 (4.9)	
	Suture needle	22 (7.7)	10 (9.5)	12 (6.6)	
	Scalpel or blade	18 (6.3)	5 (4.8)	13 (7.1)	
	Broken glass or ampoule	17 (5.9)	7 (6.7)	10 (5.5)	
Activity at time of injury	Giving an injection or inserting a cannula	69 (24.0)	30 (28.6)	39 (21.4)	0.205
	Drawing blood	54 (18.8)	18 (17.1)	36 (19.8)	
	Recapping a needle	43 (15.0)	20 (19.0)	23 (12.6)	
	Disposing of a sharp into a container	34 (11.8)	7 (6.7)	27 (14.8)	
	Handling or transporting used equipment or linen	31 (10.8)	12 (11.4)	19 (10.4)	
	Other	21 (7.3)	7 (6.7)	14 (7.7)	
	Cleaning up after a procedure	19 (6.6)	4 (3.8)	15 (8.2)	
	Assisting another staff member during a procedure	16 (5.6)	7 (6.7)	9 (4.9)	
Gloves worn at time of injury	Yes, double gloves	9 (3.1)	4 (3.8)	5 (2.7)	0.560
	Yes, single gloves	161 (56.1)	55 (52.4)	106 (58.2)	
	No	117 (40.8)	46 (43.8)	71 (39.0)	
Most recent mucocutaneous exposure (overall *n* = 198; reported all *n* = 73; underreported *n* = 125)					
Body site exposed	Eyes or conjunctiva	81 (40.9)	28 (38.4)	53 (42.4)	0.390
	Nose or mouth	72 (36.4)	24 (32.9)	48 (38.4)	
	Broken or damaged skin	34 (17.2)	17 (23.3)	17 (13.6)	
	More than one site	11 (5.6)	4 (5.5)	7 (5.6)	
Most recent exposure: source patient and shift timing (overall *n* = 392; reported all *n* = 160; underreported *n* = 232)					
Source-patient infectious status	Not known at the time	174 (44.4)	63 (39.4)	111 (47.8)	0.021
	Yes, known to be negative	96 (24.5)	34 (21.2)	62 (26.7)	
	Yes, known to be positive for a bloodborne pathogen (HIV, HBV, or HCV)	90 (23.0)	49 (30.6)	41 (17.7)	
	The source patient could not be identified	32 (8.2)	14 (8.8)	18 (7.8)	
Point in shift when exposure occurred	Second half of the shift	161 (41.1)	70 (43.8)	91 (39.2)	0.279
	First half of the shift	134 (34.2)	55 (34.4)	79 (34.1)	
	During overtime beyond my scheduled hours	59 (15.1)	25 (15.6)	34 (14.7)	
	Do not recall	38 (9.7)	10 (6.2)	28 (12.1)	
Contributing factors to the most recent exposure (overall *n* = 392; reported all *n* = 160; underreported *n* = 232)					
Rushing due to heavy workload	Very much/extremely	102 (26.0)	38 (23.8)	64 (27.6)	0.463
	Mean (SD), 1–5 scale	2.6 (1.3)	2.4 (1.3)	2.6 (1.3)	0.079
Fatigue or extended shift	Very much/Extremely	88 (22.4)	35 (21.9)	53 (22.8)	0.918
	Mean (SD), 1–5 scale	2.5 (1.2)	2.4 (1.2)	2.5 (1.2)	0.197
Patient moved unexpectedly or was uncooperative	Very much/Extremely	59 (15.1)	19 (11.9)	40 (17.2)	0.188
	Mean (SD), 1–5 scale	2.3 (1.1)	2.1 (1.1)	2.4 (1.1)	0.011
Safety-engineered devices unavailable	Very much/Extremely	59 (15.1)	13 (8.1)	46 (19.8)	0.002
	Mean (SD), 1–5 scale	2.1 (1.2)	1.8 (1.0)	2.3 (1.2)	<0.001
Sharps containers full, absent, or out of reach	Very much/Extremely	57 (14.5)	13 (8.1)	44 (19.0)	0.004
	Mean (SD), 1–5 scale	2.2 (1.2)	1.9 (1.0)	2.4 (1.2)	<0.001
PPE unavailable or ill-fitting	Very much/Extremely	68 (17.3)	22 (13.8)	46 (19.8)	0.154
	Mean (SD), 1–5 scale	2.3 (1.1)	2.1 (1.1)	2.4 (1.1)	<0.001
Did not follow correct procedure	Very much/Extremely	60 (15.3)	20 (12.5)	40 (17.2)	0.255
	Mean (SD), 1–5 scale	2.3 (1.1)	2.2 (1.1)	2.3 (1.1)	0.111

Data are *n* (%) unless otherwise indicated. Percentages use the denominator of nurses to whom each question applies: all 392 exposed nurses for exposure frequency, source-patient characteristics, shift timing, and contributing factors; 287 nurses with at least one percutaneous injury for device, activity, and gloves; and 198 nurses with at least one mucocutaneous exposure for body site. Group denominators therefore differ accordingly (percutaneous at-risk: 105 reported all, 182 underreported; mucocutaneous at-risk: 73 reported all, 125 underreported). Underreported = failed to formally report at least one exposure in the past 12 months. *p*-values were calculated using Pearson’s chi-square test or Fisher’s exact test when any expected cell count was below 5; the Wilcoxon rank-sum test was used for contributing-factor scores. No *p*-values are reported for percutaneous injury frequency, mucocutaneous exposure frequency, total exposure counts, or exposures per nurse because a greater number of exposures mechanically increases the opportunity for classification as underreported; these rows are presented descriptively only. *p*-values are descriptive and are not adjusted for multiple comparisons. IQR, interquartile range; PPE, personal protective equipment; SD, standard deviation.

Secondary outcomes comprised the aggregate proportions of exposure counts reported and unreported; reporting of the most recent exposure; the interval from the most recent reported exposure to notification; nurse-level receipt of immediate wound care or irrigation advice, baseline serology, hepatitis B immunoglobulin or a vaccine booster, HIV PEP, and counseling or psychological support; and PEP completion among nurses advised to undertake it. Post-exposure-management questions assessed whether each service was received at least once after any BBF exposure during the full 12-month recall period; they neither referred exclusively to the most recent exposure nor linked services to individual exposure counts. A partial reporter could therefore have received care after one formally reported exposure while leaving another unreported. The clinical indication for each intervention also could not be determined because it depends on exposure route, source-patient status, hepatitis B vaccination and immune status, and other clinical risk-assessment findings. Device, activity, PPE use, anatomical site, source-patient status, shift timing, and contributing factors referred only to the most recent applicable exposure. These characteristics could not be assigned to all 747 exposure counts or used to determine how many of the 373 unreported counts involved a pathogen-positive or unknown source, clinically meaningful transmission risk, or an indication for prophylaxis.

The adjustment set included age per 5 years; professional rank, classified as junior or senior staff nurse *versus* charge nurse or supervisor; clinical area, grouped by higher, moderate, or lower expected exposure risk; permanent *versus* non-permanent employment; night-shift frequency; biosafety training during the preceding 12 months; and full or general *versus* vague or absent knowledge of the reporting procedure. Higher-exposure areas comprised intensive care, emergency, isolation or high-consequence-pathogen units, and operating or procedure rooms; moderate-exposure areas comprised general infectious disease wards and dialysis; and lower-exposure areas comprised outpatient or fever clinics and other areas. Night-shift frequency was entered as an ordinal score (0–3), and its linearity was evaluated against a categorical specification. Years as a nurse and years at the hospital were not included in adjusted models because of conceptual and empirical overlap with age and professional rank, and usual workload was not included because of overlap with night-shift frequency and clinical area. Collinearity was assessed using variance-inflation factors. Knowledge of PEP access and confidence in anonymity were regarded as proximal reporting-context variables rather than baseline confounders and were examined in unadjusted analyses only.

Safety climate, psychological safety, and perceived organizational support were treated as concurrently measured individual perceptions, not institution-level exposures. Safety climate and perceived organizational support were included in Model 1, and psychological safety was added in Model 2 to assess the robustness of these associations after accounting for shared variation. Collinearity was evaluated using variance-inflation factors, condition indices, and changes in coefficient precision across models. Models containing these scales were not interpreted as estimating direct, indirect, or mediated effects. Branching responses recorded as “not applicable” for respondents who were not routed to a question were excluded from the relevant denominator rather than treated as substantive categories. Completed questionnaires contained no missing values; therefore, no imputation was undertaken.

### 2.5 Statistical analysis

Categorical variables were summarized as frequencies and percentages, while continuous variables were summarized using mean (SD) or median (IQR), according to their distribution. The 95% CI for the prevalence of nurse-level underreporting was calculated using the Wilson score method (21). Exposed and unexposed nurses and, within the exposed cohort, complete reporters and under-reporters were compared using Pearson’s *χ*^2^ test or Fisher’s exact test when any expected cell count was <5. Continuous variables and Likert-scored items were compared using the Wilcoxon rank-sum test. Likert items were summarized using their complete 1–5 distributions and the proportion selecting “very much” or “extremely.” No significance tests were applied to comparisons structurally determined by the underreporting definition, including exposure frequency, aggregate reported and unreported counts, categorical reporting proportions, and reporting of the most recent exposure, because additional exposures mechanically increase the opportunity for classification as an under-reporter.

The primary association analysis used modified Poisson regression with an HC0 robust sandwich variance estimator to model whether an exposed nurse had at least one unreported exposure, producing prevalence ratios with 95% CIs (22). Model 1 adjusted for age per 5 years, professional rank, clinical area, employment status, night-shift frequency, biosafety training, knowledge of the reporting procedure, safety climate, and perceived organizational support; Model 2 additionally included psychological safety. Covariates were retained on substantive grounds without selection by univariable *p*-values or automated stepwise procedures. Because this outcome is structurally dependent on the number of exposure opportunities, its estimates were interpreted as associations with the prevalence of nurses affected by any underreporting, not with the probability that an individual exposure remained unreported.

Exposure opportunity and distributional assumptions were examined in prespecified sensitivity analyses. Negative-binomial regression modeled unreported exposure counts with the logarithm of total exposures as an offset, producing incidence rate ratios (IRRs); because this model does not formally constrain unreported counts to the total experienced, it was interpreted as supportive rather than definitive. A hurdle model combined a binomial component with a logit link comparing nurses with and without any unreported exposure, producing odds ratios (ORs), with a zero-truncated negative-binomial component with a log link modeling unreported counts among under-reporters, producing IRRs. Because its binary component closely reproduced the nurse-level outcome, the hurdle model was interpreted solely as a distributional sensitivity analysis and not as evidence of a two-stage reporting mechanism or behavioral threshold. Firth penalized logistic regression provided a small-sample and separation check (23), and the ordinal specification of night-shift frequency was compared with a categorical specification.

Internal consistency of the organizational scales was assessed using ordinal Cronbach’s α and McDonald’s ω derived from polychoric correlation matrices. Confirmatory factor analysis treated the five-category Likert responses as ordered categorical variables and used robust diagonally weighted least-squares estimation with mean- and variance-adjusted test statistics. Model fit was evaluated using the comparative fit index, Tucker–Lewis index, root mean square error of approximation with its 90% CI, and standardized root mean square residual (24). Supplementary Table S2 reports reliability coefficients, fit indices, item-level loadings from confirmatory and exploratory (promax-rotated) factor analyses, measurement invariance, and correlations among scale scores.

Single-factor models were fitted separately for psychological safety, safety climate, and perceived organizational support. Psychological safety measurement invariance across permanent (*n* = 431) and non-permanent employees (*n* = 441) was assessed using sequential configural and metric models fitted with the same ordinal estimator; a reduction in the comparative fit index (CFI) of ≤0.01 was considered consistent with invariant factor loadings (25). The prespecified five-factor structure of the 12-item reasons inventory was evaluated among 232 under-reporters using the ordinal estimator, with loadings constrained to equality within the three two-item factors for identification. Because conventional fit criteria were not met, no factor scores, subscale scores, or domain-level inferences were calculated. Reasons were analyzed individually, conceptual groupings were used only as descriptive organizational labels, and arithmetic combinations of selected items were not interpreted as validated domains.

Formal mediation analysis was not undertaken because safety climate, psychological safety, and reporting behavior were measured concurrently, precluding the temporal ordering required for mediation inference (45). Consequently, no direct, indirect, or mediated effects or proportions mediated were estimated. No adjustment for multiple comparisons was applied because the analyses were exploratory and estimation-oriented (26). Effect estimates and 95% CIs were emphasized, and *p*-values were interpreted as continuous measures of compatibility rather than against a binary threshold. All tests were two-sided (*α* = 0.05). Analyses were conducted in R version 4.4.3 using the sandwich and lmtest packages for robust inference, MASS and pscl for count and hurdle models, logistf for penalized regression, psych and lavaan for psychometric analyses, and ggplot2 for figures.

## 3 Results

### 3.1 Participant characteristics

Of 1,110 eligible nurses invited, 872 returned a complete questionnaire (response rate 78.6%; Figure 1). The cohort was young and predominantly female (mean age 31.8 years, SD 7.4; 825 women, 94.6%); 550 (63.1%) occupied junior or senior staff nurse positions, and 441 (50.6%) were employed on non-permanent terms (Table 2). Workload was substantial: 519 (59.5%) worked five or more night shifts per month, 367 (42.1%) described their usual shift as heavy or very heavy, and 383 (43.9%) often worked while physically exhausted. Baseline protection was largely in place, but procedural preparedness was not: although 773 (88.6%) had completed a full hepatitis B course and 745 (85.4%) had received biosafety training in the preceding 12 months, 363 (41.6%) had only a vague idea of the reporting procedure or did not know it at all, and 169 (19.4%) knew neither the location of the PEP service nor how to access it out of hours. Mean scores on the three organizational scales were near the midpoint of the 1–5 range (psychological safety 3.0, safety climate 3.3, perceived organizational support 3.3; SD 0.9–1.0).

**Figure 1:**
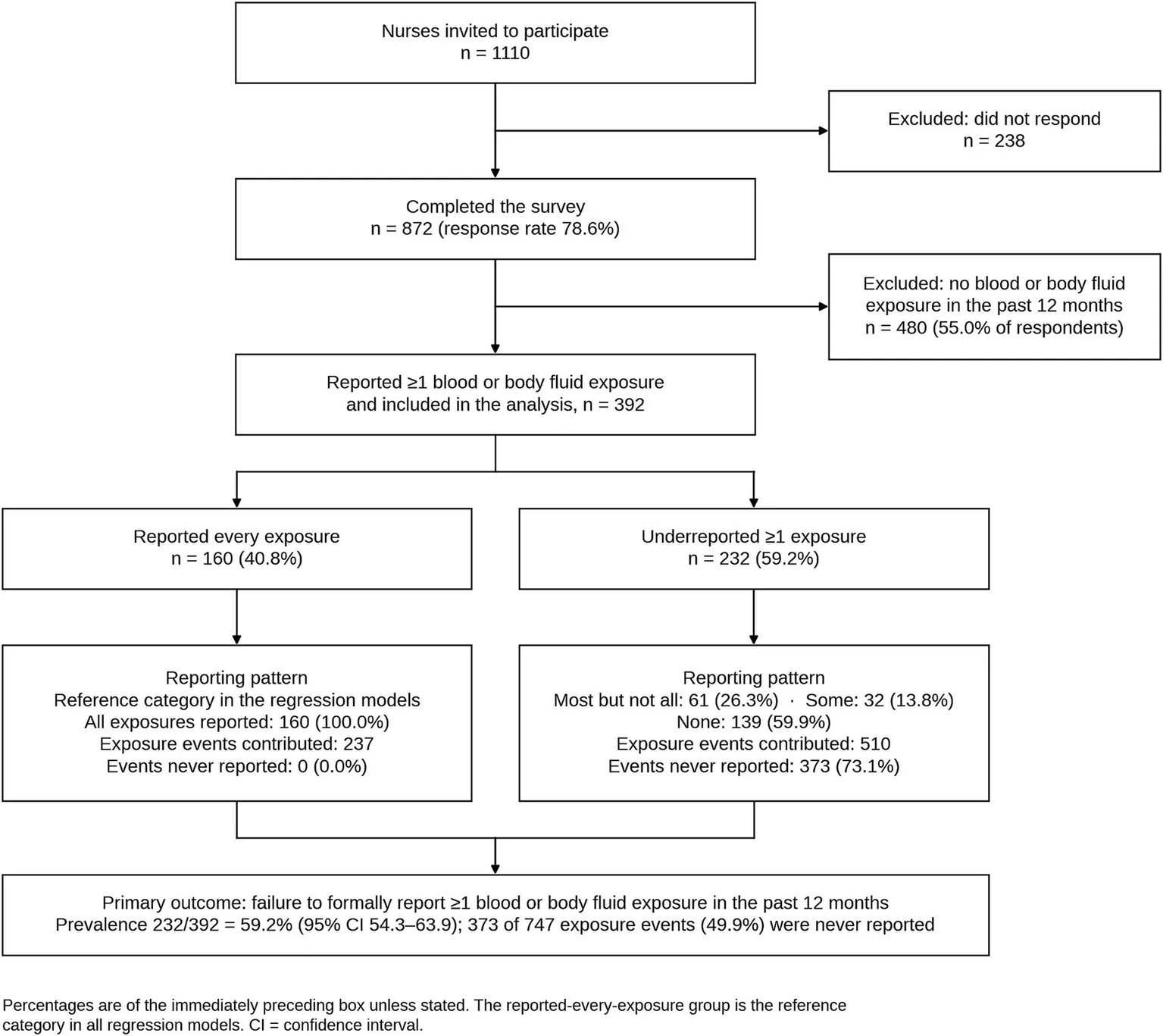
Flow of participants and derivation of the primary outcome. The numbers of eligible nurses invited and respondents, as well as the exclusion of those without blood or body fluid exposure in the preceding 12 months, are shown, leaving the exposed cohort. Reporting pattern, exposures contributed, and exposures not reported are shown for complete reporters and under-reporters. Exposure totals are aggregate respondent-reported counts. Percentages refer to the preceding box unless stated; complete reporters form the reference category in the nurse-level regression models. CI, confidence interval.

**Table 2:** Characteristics of participating nurses.

Variable	Category	Overall (*N* = 872)	Unexposed (*n* = 480)	Exposed (*n* = 392)	*p*
Demographic and professional characteristics					
Age (years), mean (SD)		31.8 (7.4)	33.0 (8.0)	30.4 (6.3)	<0.001
Sex	Female	825 (94.6)	451 (94.0)	374 (95.4)	0.335
	Male	44 (5.0)	26 (5.4)	18 (4.6)	
	Prefer not to say	3 (0.3)	3 (0.6)	0 (0.0)	
Highest nursing qualification	Diploma in nursing	245 (28.1)	144 (30.0)	101 (25.8)	0.388
	Bachelor’s degree (BSN or equivalent)	589 (67.5)	316 (65.8)	273 (69.6)	
	Master’s degree or higher	32 (3.7)	18 (3.8)	14 (3.6)	
	Other	6 (0.7)	2 (0.4)	4 (1.0)	
Professional rank	Staff nurse/junior nurse	203 (23.3)	91 (19.0)	112 (28.6)	<0.001
	Senior staff nurse	347 (39.8)	185 (38.5)	162 (41.3)	
	Charge nurse/ward in-charge	247 (28.3)	144 (30.0)	103 (26.3)	
	Nursing supervisor or above	75 (8.6)	60 (12.5)	15 (3.8)	
Total years as a nurse	Less than 2 years	125 (14.3)	49 (10.2)	76 (19.4)	<0.001
	2–5 years	161 (18.5)	88 (18.3)	73 (18.6)	
	6–10 years	224 (25.7)	113 (23.5)	111 (28.3)	
	More than 10 years	362 (41.5)	230 (47.9)	132 (33.7)	
Years at this hospital	Less than 2 years	167 (19.2)	67 (14.0)	100 (25.5)	<0.001
	2–5 years	207 (23.7)	113 (23.5)	94 (24.0)	
	6–10 years	255 (29.2)	139 (29.0)	116 (29.6)	
	More than 10 years	243 (27.9)	161 (33.5)	82 (20.9)	
Employment status	Permanent/regular staff	431 (49.4)	253 (52.7)	178 (45.4)	0.007
	Contract staff	343 (39.3)	185 (38.5)	158 (40.3)	
	Agency, deputed, or temporary staff	52 (6.0)	27 (5.6)	25 (6.4)	
	Intern or nurse in training	46 (5.3)	15 (3.1)	31 (7.9)	
Main clinical area	General infectious disease ward	264 (30.3)	155 (32.3)	109 (27.8)	0.362
	Isolation or high-consequence pathogen unit	123 (14.1)	68 (14.2)	55 (14.0)	
	Intensive care unit	111 (12.7)	58 (12.1)	53 (13.5)	
	Emergency department	100 (11.5)	44 (9.2)	56 (14.3)	
	Outpatient or fever clinic	97 (11.1)	58 (12.1)	39 (9.9)	
	Dialysis unit	73 (8.4)	40 (8.3)	33 (8.4)	
	Operating room or procedure suite	56 (6.4)	30 (6.2)	26 (6.6)	
	Other	48 (5.5)	27 (5.6)	21 (5.4)	
Night shifts per month	No night shifts	116 (13.3)	85 (17.7)	31 (7.9)	<0.001
	1–4 per month	237 (27.2)	136 (28.3)	101 (25.8)	
	5–8 per month	360 (41.3)	190 (39.6)	170 (43.4)	
	More than 8 per month	159 (18.2)	69 (14.4)	90 (23.0)	
Patients per shift	Fewer than 5	138 (15.8)	79 (16.5)	59 (15.1)	0.017
	5–9	298 (34.2)	173 (36.0)	125 (31.9)	
	10–14	276 (31.7)	158 (32.9)	118 (30.1)	
	15 or more	160 (18.3)	70 (14.6)	90 (23.0)	
Vaccination, training, and knowledge					
Hepatitis B vaccination	Completed the full course (3 or more doses)	773 (88.6)	426 (88.8)	347 (88.5)	0.616
	Received 1 or 2 doses only	48 (5.5)	29 (6.0)	19 (4.8)	
	Never vaccinated	24 (2.8)	13 (2.7)	11 (2.8)	
	Not sure	27 (3.1)	12 (2.5)	15 (3.8)	
Biosafety training (past 12 months)	Yes, more than once	433 (49.7)	234 (48.8)	199 (50.8)	0.778
	Yes, once	312 (35.8)	173 (36.0)	139 (35.5)	
	No	127 (14.6)	73 (15.2)	54 (13.8)	
Knowledge of reporting procedure	I know the full procedure and could follow it without help	230 (26.4)	135 (28.1)	95 (24.2)	0.618
	I know the general steps but would need to check some details	279 (32.0)	149 (31.0)	130 (33.2)	
	I have only a vague idea of the procedure	245 (28.1)	131 (27.3)	114 (29.1)	
	I do not know the procedure at all	118 (13.5)	65 (13.5)	53 (13.5)	
Knowledge of PEP location and access	Yes, both	393 (45.1)	221 (46.0)	172 (43.9)	0.075
	I know the location but not the after-hours access	310 (35.6)	179 (37.3)	131 (33.4)	
	I know neither	169 (19.4)	80 (16.7)	89 (22.7)	
Workload, perceptions, and organizational climate					
Usual workload during a shift	Very light	91 (10.4)	66 (13.8)	25 (6.4)	<0.001
	Light	183 (21.0)	118 (24.6)	65 (16.6)	
	Moderate	231 (26.5)	152 (31.7)	79 (20.2)	
	Heavy	226 (25.9)	89 (18.5)	137 (34.9)	
	Very heavy	141 (16.2)	55 (11.5)	86 (21.9)	
Work while physically exhausted	Never	89 (10.2)	61 (12.7)	28 (7.1)	<0.001
	Rarely	174 (20.0)	114 (23.8)	60 (15.3)	
	Sometimes	226 (25.9)	131 (27.3)	95 (24.2)	
	Often	220 (25.2)	104 (21.7)	116 (29.6)	
	Almost every shift	163 (18.7)	70 (14.6)	93 (23.7)	
Work while unwell (past 3 months)	Never	284 (32.6)	172 (35.8)	112 (28.6)	0.013
	Once	208 (23.9)	122 (25.4)	86 (21.9)	
	2–3 times	167 (19.2)	88 (18.3)	79 (20.2)	
	4–5 times	126 (14.4)	61 (12.7)	65 (16.6)	
	More than 5 times	87 (10.0)	37 (7.7)	50 (12.8)	
Full PPE makes procedures harder	Strongly disagree	66 (7.6)	38 (7.9)	28 (7.1)	0.318
	Disagree	176 (20.2)	105 (21.9)	71 (18.1)	
	Neutral	263 (30.2)	149 (31.0)	114 (29.1)	
	Agree	255 (29.2)	134 (27.9)	121 (30.9)	
	Strongly agree	112 (12.8)	54 (11.2)	58 (14.8)	
Perceived personal risk of infection	Very low	76 (8.7)	67 (14.0)	9 (2.3)	<0.001
	Low	200 (22.9)	128 (26.7)	72 (18.4)	
	Moderate	243 (27.9)	139 (29.0)	104 (26.5)	
	High	233 (26.7)	107 (22.3)	126 (32.1)	
	Very high	120 (13.8)	39 (8.1)	81 (20.7)	
Confidence in response anonymity	Not at all confident	124 (14.2)	56 (11.7)	68 (17.3)	0.033
	Slightly confident	174 (20.0)	98 (20.4)	76 (19.4)	
	Moderately confident	218 (25.0)	117 (24.4)	101 (25.8)	
	Very confident	202 (23.2)	110 (22.9)	92 (23.5)	
	Completely confident	154 (17.7)	99 (20.6)	55 (14.0)	
Likelihood of reporting next exposure	Very unlikely	121 (13.9)	41 (8.5)	80 (20.4)	<0.001
	Unlikely	146 (16.7)	72 (15.0)	74 (18.9)	
	Unsure	188 (21.6)	106 (22.1)	82 (20.9)	
	Likely	212 (24.3)	124 (25.8)	88 (22.4)	
	Very likely	205 (23.5)	137 (28.5)	68 (17.3)	
Psychological safety score, mean (SD)		3.0 (0.9)	3.1 (0.8)	2.9 (0.9)	0.001
Safety climate score, mean (SD)		3.3 (0.9)	3.4 (0.8)	3.2 (0.9)	<0.001
Perceived organizational support, mean (SD)		3.3 (1.0)	3.3 (0.9)	3.1 (1.0)	0.005

Values are *n* (%) unless otherwise indicated. *p*-values were calculated using the chi-square test or Fisher’s exact test for categorical variables and the Wilcoxon rank-sum test for continuous variables. No multiplicity adjustment was applied; *p*-values are presented as measures of imbalance rather than for inferential testing. Psychological safety, safety climate, and perceived organizational support scores range from 1 to 5, with higher scores indicating more positive perceptions. SD, standard deviation; PEP, post-exposure prophylaxis; PPE, personal protective equipment.

Exposed and unexposed nurses differed in work organization rather than in training or credentials: the exposed group was younger (30.4 vs. 33.0 years), more junior, more often non-permanent, worked more night shifts and reported heavier night-shift workloads, and scored lower on all three scales (*p* ≤ 0.007) but was indistinguishable on sex, qualification, clinical area, vaccination, training, and procedural knowledge (*p* ≥ 0.335).

### 3.2 Exposure burden and circumstances

In the preceding 12 months, 392 of 872 nurses (45.0, 95% CI 41.6–48.3) reported at least one BBF exposure, accounting for 747 events (Figure 1). Percutaneous injury predominated: 287 nurses (32.9% of the cohort; 73.2% of the exposed) reported 458 sharps injuries, and 198 (22.7%; 50.5%) reported 289 mucocutaneous exposures, with 93 (23.7%) sustaining both. Exposure was rarely a single event (mean 1.9 per exposed nurse, SD 1.1; median 2, IQR 1–2; maximum 6). Nurses classified as under-reporters reported a mean of 2.2 exposures compared with 1.5 among complete reporters. This comparison is descriptive and was not subjected to interprested inferentially because a larger exposure count mechanically provides more opportunities to meet the definition of having at least one unreported exposure.

Among the 287 most recent percutaneous injuries, the commonest devices were hollow-bore hypodermic needles (69, 24.0%) and intravenous cannulas (55, 19.2%), the commonest activity was injection or cannulation (69, 24.0%), and no gloves were worn in 117 (40.8%); none differed by reporting status (Table 1). For the most recent exposure, source status was unknown at the time for 174 nurses (44.4%) and the source patient could not be identified for 32 (8.2%), while 90 (23.0%) reported a source known to be positive for HIV, hepatitis B, or hepatitis C; the distribution of source status differed by reporting status (known positive: 30.6% of complete reporters vs. 17.7% of under-reporters; *p* = 0.021). Most recent exposures occurred most often in the second half of the shift (161, 41.1%), and 59 (15.1%) occurred during unscheduled overtime.

Contributing factors differed between the groups, but the findings depended on the scaling used. Dichotomized at “very much or extremely,” only unavailable safety-engineered devices (19.8% of under-reporters vs. 8.1% of full reporters; *p* = 0.002) and full, absent, or unreachable sharps containers (19.0% vs. 8.1%; *p* = 0.004) differed; on the full 1–5 scale, these were joined by ill-fitting or unavailable protective equipment (2.4 vs. 2.1, *p* < 0.001; dichotomy *p* = 0.154) and unexpected patient movement (2.4 vs. 2.1, *p* = 0.011; dichotomy *p* = 0.188). Rushing, fatigue, and procedural non-adherence differed on neither scale.

### 3.3 Underreporting prevalence and the reporting cascade

Among the 392 exposed nurses, 232 failed to report at least one exposure, an underreporting prevalence of 59.2% (95% CI 54.3–63.9) (Table 3; Figure 1). On the self-reported categorical item, 160 (40.8%) reported every exposure, 61 (15.6%) reported most, 32 (8.2%) reported some, and 139 (35.5%) reported none; because nurses with a single exposure could only report all or none, this distribution was not interpreted as evidence of a behavioral threshold. Across aggregate exposure counts, 374 of 747 (50.1%) were formally reported, and 373 (49.9%) were not. Complete reporters contributed 237 exposures, all reported, whereas under-reporters contributed 510, of which 373 (73.1%) were not reported (mean 1.61 unreported exposures per under-reporter, SD 0.88; maximum 6). The most recent exposure had never been reported by 197 nurses (50.3% of the exposed; 84.9% of under-reporters, and none of the complete reporters). Where the most recent exposure was reported, reporting was generally prompt, although few under-reporters contributed to this comparison. Of the 195 nurses whose most recent exposure was reported, 54 (27.7%) reported within 1 h and 173 (88.7%) within 24 h, with no group difference in the reporting interval (*p* = 0.759) or reporting within 24 h (*p* = 0.771).

**Table 3:** Reporting behavior and post-exposure management, overall and by reporting status.

Variable	Category	Overall, *n* (%)	Reported all, *n* (%)	Underreported, *n* (%)	*p*-value
Proportion of exposures formally reported in the past 12 months^a^ (overall *n* = 392; reported all *n* = 160; underreported *n* = 232)					
Self-reported reporting behavior	Reported all exposures	160 (40.8)	—	—	—
	Reported most but not all	61 (15.6)	—	—	
	Reported some	32 (8.2)	—	—	
	Reported none	139 (35.5)	—	—	
Primary outcome	Underreported (≥1 exposure not reported)	232 (59.2; 95% CI 54.3–63.9)	—	—	—
	Reported all exposures	160 (40.8)	—	—	
Aggregate exposure counts^a^ (total events *n* = 747; reported all *n* = 237; underreported *n* = 510)					
Exposure counts	Total exposures contributed	747	237	510	—
	Exposures formally reported	374 (50.1)	237 (100.0)	137 (26.9)	
	Exposures not reported	373 (49.9)	0 (0.0)	373 (73.1)	
Most recent exposure: reporting status^a^ (overall *n* = 392; reported all *n* = 160; underreported *n* = 232)					
Was the most recent exposure reported	Yes, reported by the nurse	175 (44.6)	143 (89.4)	32 (13.8)	—
	Yes, reported by someone else	20 (5.1)	17 (10.6)	3 (1.3)	
	No, never formally reported	197 (50.3)	0 (0.0)	197 (84.9)	
Timeliness of reporting, among nurses whose most recent exposure was reported^b^ (overall *n* = 195; reported all *n* = 160; underreported *n* = 35)					
Interval from exposure to report	Within 1 h	54 (27.7)	46 (28.8)	8 (22.9)	0.759
	Within 2–8 h	68 (34.9)	53 (33.1)	15 (42.9)	
	Within 9–24 h	51 (26.2)	42 (26.2)	9 (25.7)	
	More than 24 h later	22 (11.3)	19 (11.9)	3 (8.6)	
Reported within 24 h	Yes	173 (88.7)	141 (88.1)	32 (91.4)	0.771
Post-exposure services received (multiple response)^c^ (overall *n* = 392; reported all *n* = 160; underreported *n* = 232)					
Immediate wound care or irrigation advice	Received	263 (67.1)	145 (90.6)	118 (50.9)	<0.001
Baseline blood testing or serology	Received	162 (41.3)	134 (83.8)	28 (12.1)	<0.001
Hepatitis B immunoglobulin or vaccine booster	Received	58 (14.8)	49 (30.6)	9 (3.9)	<0.001
HIV post-exposure prophylaxis	Received	43 (11.0)	38 (23.8)	5 (2.2)	<0.001
Counseling or psychological support	Received	62 (15.8)	54 (33.8)	8 (3.4)	<0.001
None of the above	Received	113 (28.8)	1 (0.6)	112 (48.3)	<0.001
Number of services received	Mean (SD), 0–5	1.5 (1.3)	2.6 (0.9)	0.7 (0.9)	<0.001
	Median [IQR]	1.0 [0.0–3.0]	3.0 [2.0–3.0]	1.0 [0.0–1.0]	—
Post-exposure prophylaxis: advised^c^ (overall *n* = 392; reported all *n* = 160; underreported *n* = 232)					
Advised to take post-exposure prophylaxis	Yes	182 (46.4)	151 (94.4)	31 (13.4)	<0.001
Post-exposure prophylaxis: completion among those advised^c^ (overall *n* = 182; reported all *n* = 151; underreported *n* = 31)					
Completion of the advised course	Completed in full	115 (63.2)	93 (61.6)	22 (71.0)	0.470
	Started but did not complete	45 (24.7)	40 (26.5)	5 (16.1)	
	Never started	22 (12.1)	18 (11.9)	4 (12.9)	
Reasons for not completing post-exposure prophylaxis^d^ (overall *n* = 67; reported all *n* = 58; underreported *n* = 9)					
Main reason given	Too busy or could not leave the ward	20 (29.9)	18 (31.0)	2 (22.2)	0.765
	Believed the risk was too low	15 (22.4)	12 (20.7)	3 (33.3)	
	Side effects of the medication	13 (19.4)	12 (20.7)	1 (11.1)	
	Concerned others would find out	8 (11.9)	7 (12.1)	1 (11.1)	
	Follow-up appointments inconvenient	7 (10.4)	5 (8.6)	2 (22.2)	
	Other	4 (6.0)	4 (6.9)	0 (0.0)	

Data are *n* (%) unless otherwise indicated. Denominators are stated in each section header and differ between sections because several items are skip-patterned; group denominators differ accordingly.

a These rows are definitional or structurally determined by the primary outcome, so testing them against that outcome would be circular, and no *p*-value is reported. Nurses who reported all exposures necessarily reported their most recent exposure and necessarily contributed no unreported exposures; group columns are omitted where every cell would be 0% or 100%. Exposure totals are aggregate respondent-reported counts, not individually characterized exposure records.

b Timeliness applies only to the 195 nurses whose most recent exposure was formally reported. All 160 nurses who reported every exposure fall in this subgroup, but only 35 under-reporters do, so the comparison is underpowered and the corresponding *p*-value is exploratory.

c Post-exposure services were measured at the respondent level as receipt of each service at least once following any exposure during the 12-month recall period. Services were not linked to individual reported or unreported exposures, and these comparisons describe nurse-level service receipt according to reporting pattern. An under-reporter may have received a service after a different exposure that was formally reported, and clinical indications for each service could not be determined.

d Reasons are reported for the 67 nurses advised to take prophylaxis who did not complete it (58 who reported all exposures and 9 under-reporters). The under-reporter subgroup is too small for a stable comparison, and the corresponding *p*-value is exploratory. *p*-Values were calculated using Pearson’s chi-square test or Fisher’s exact test when any expected cell count was below 5; the Wilcoxon rank-sum test was used for the number of services received. *p*-Values are descriptive and are not corrected for multiple comparisons. IQR, interquartile range; PEP, post-exposure prophylaxis; SD, standard deviation.

### 3.4 Post-exposure care received

Under-reporters reported substantially lower receipt of each post-exposure service at the nurse level. These comparisons indicate whether a service was received at least once during the recall period and do not provide one-to-one linkage between an individual unreported exposure and the care subsequently received. Accordingly, these findings describe a nurse-level care deficit associated with underreporting rather than the event-specific causal effect of non-reporting (Table 3). Comparing complete reporters with under-reporters, wound care or irrigation advice was received by 145 of 160 (90.6%) *versus* 118 of 232 (50.9%), baseline serology by 83.8% *versus* 12.1%, hepatitis B immunoglobulin or a booster by 30.6% *versus* 3.9%, HIV PEP by 23.8% *versus* 2.2%, and counseling by 33.8% *versus* 3.4% (all *p* < 0.001); the mean number of the five services received was 2.6 *versus* 0.7 (*p* < 0.001), and 112 under-reporters (48.3%) received none of the five services, compared with 1 complete reporter (0.6%). PEP was advised for 151 complete reporters (94.4%) and 31 under-reporters (13.4%; *p* < 0.001), and among those advised, 115 of 182 (63.2%) completed the course, with no material difference between groups (*p* = 0.470).

### 3.5 Correlates of underreporting

Unadjusted and adjusted prevalence ratios are given in Table 4 and the primary adjusted model in Figure 2. In unadjusted analyses, underreporting fell with age (PR 0.83 per 5 years, 95% CI 0.76–0.90), seniority (0.71, 0.60–0.83), and each organizational scale per standard deviation (0.80, 0.74–0.87; 0.76, 0.71–0.82; and 0.90, 0.83–0.97, respectively), and rose with non-permanent employment (1.53, 1.26–1.85), not knowing where or when PEP could be accessed (1.63, 1.34–1.97), and very heavy workload (3.78, 1.71–8.35).

**Table 4:** Factors associated with underreporting of blood and body fluid exposures: unadjusted and adjusted prevalence ratios.

Variable	Unadjusted PR (95% CI)	*p*	Model 1 aPR (95% CI)	*p*	Model 2 aPR (95% CI)	*p*
Age (per 5 years)	0.83 (0.76–0.90)	<0.001	0.96 (0.86–1.07)	0.441	0.96 (0.86–1.07)	0.422
Sex (male *vs*. female) (ref: female)	1.00 (ref)		—		—	
Male	1.13 (0.81–1.59)	0.467	—		—	
Prefer not to say	—		—		—	
Education (ref: diploma in nursing)	1.00 (ref)		—		—	
Bachelor’s degree (BSN or equivalent)	1.20 (0.97–1.48)	0.087	—		—	
Master’s degree or higher	1.11 (0.68–1.81)	0.678	—		—	
Other	1.46 (0.80–2.65)	0.217	—		—	
Professional rank (collapsed) (ref: junior/senior staff nurse)	1.00 (ref)		1.00 (ref)		1.00 (ref)	
Charge/Supervisor (vs. junior/senior)	0.71 (0.60–0.83)	<0.001	0.97 (0.74–1.27)	0.845	0.97 (0.74–1.27)	0.819
Total years as a nurse (ref: less than 2 years)	1.00 (ref)		—		—	
2–5 years	0.86 (0.71–1.03)	0.100	—		—	
6–10 years	0.70 (0.57–0.84)	<0.001	—		—	
More than 10 years	0.52 (0.41–0.65)	<0.001	—		—	
Years at this hospital (ref: less than 2 years)	1.00 (ref)		—		—	
2–5 years	0.75 (0.62–0.90)	0.003	—		—	
6–10 years	0.64 (0.52–0.78)	<0.001	—		—	
More than 10 years	0.51 (0.39–0.67)	<0.001	—		—	
Employment (collapsed) (ref: permanent)	1.00 (ref)		1.00 (ref)		1.00 (ref)	
Non-permanent (*vs*. permanent)	1.53 (1.26–1.85)	<0.001	1.30 (1.07–1.57)	0.007	1.29 (1.07–1.55)	0.008
Department (collapsed) (ref: high-exposure)	1.00 (ref)		1.00 (ref)		1.00 (ref)	
Moderate-exposure	—		0.98 (0.83–1.15)	0.763	1.00 (0.85–1.18)	0.960
Lower-exposure	—		1.06 (0.83–1.36)	0.638	1.07 (0.83–1.37)	0.593
Night shifts per month (ref: no night shifts; adjusted models: ordinal score per level)	1.00 (ref)		1.14 (1.04–1.25)	0.004	1.16 (1.05–1.27)	0.002
1–4 per month	1.25 (0.82–1.91)	0.303				
5–8 per month	1.35 (0.90–2.03)	0.143				
More than 8 per month	1.40 (0.92–2.13)	0.113				
Patients per shift (ref: fewer than 5)	1.00 (ref)		—		—	
5–9	1.06 (0.80–1.40)	0.672	—		—	
10–14	1.09 (0.83–1.44)	0.527	—		—	
15 or more	1.19 (0.90–1.57)	0.228	—		—	
HBV vaccination (ref: completed full course)	1.00 (ref)		—		—	
Received 1 or 2 doses only	0.60 (0.33–1.08)	0.089	—		—	
Never vaccinated	0.44 (0.17–1.16)	0.099	—		—	
Not sure	0.86 (0.53–1.40)	0.554	—		—	
Biosafety training (collapsed) (ref: any training)	1.00 (ref)		1.00 (ref)		1.00 (ref)	
No training (*vs*. any)	1.21 (1.01–1.45)	0.036	1.03 (0.85–1.25)	0.742	1.05 (0.87–1.26)	0.639
Knowledge of procedure (collapsed) (ref: full/general knowledge)	1.00 (ref)		1.00 (ref)		1.00 (ref)	
Vague/none (vs. full/general)	1.17 (0.87–1.57)	0.296	1.56 (1.31–1.86)	<0.001	1.54 (1.29–1.84)	<0.001
Knowledge of PEP access (ref: yes, both)	1.00 (ref)		—		—	
I know the location but not the after-hours access	1.30 (1.06–1.59)	0.014	—		—	
I know neither	1.63 (1.34–1.97)	<0.001	—		—	
Usual workload (ref: very light)	1.00 (ref)		—		—	
Light	2.54 (1.12–5.76)	0.026	—		—	
Moderate	2.97 (1.33–6.65)	0.008	—		—	
Heavy	2.99 (1.35–6.63)	0.007	—		—	
Very heavy	3.78 (1.71–8.35)	0.001	—		—	
Work while exhausted (ref: never)	1.00 (ref)		—		—	
Rarely	1.13 (0.68–1.86)	0.638	—		—	
Sometimes	1.15 (0.72–1.85)	0.552	—		—	
Often	1.61 (1.03–2.51)	0.036	—		—	
Almost every shift	1.61 (1.02–2.52)	0.039	—		—	
Work while unwell (ref: never)	1.00 (ref)		—		—	
Once	1.17 (0.91–1.50)	0.235	—		—	
2–3 times	1.19 (0.93–1.54)	0.171	—		—	
4–5 times	1.36 (1.07–1.74)	0.013	—		—	
More than 5 times	1.22 (0.92–1.62)	0.172	—		—	
PPE makes procedures harder (ref: strongly disagree)	1.00 (ref)		—		—	
Disagree	1.22 (0.75–1.97)	0.427	—		—	
Neutral	1.39 (0.88–2.19)	0.153	—		—	
Agree	1.45 (0.92–2.27)	0.108	—		—	
Strongly agree	1.61 (1.01–2.55)	0.043	—		—	
Perceived risk of infection (ref: very low)	1.00 (ref)		—		—	
Low	0.95 (0.51–1.77)	0.872	—		—	
Moderate	0.93 (0.51–1.73)	0.829	—		—	
High	1.19 (0.65–2.16)	0.576	—		—	
Very high	1.16 (0.63–2.12)	0.640	—		—	
Confidence in anonymity (ref: not at all confident)	1.00 (ref)		—		—	
Slightly confident	0.97 (0.77–1.23)	0.813	—		—	
Moderately confident	0.88 (0.70–1.11)	0.269	—		—	
Very confident	0.85 (0.67–1.08)	0.190	—		—	
Completely confident	0.62 (0.43–0.88)	0.007	—		—	
Psychological safety (per 1 SD)	0.80 (0.74–0.87)	<0.001	—		0.87 (0.79–0.95)	0.001
Safety climate (per 1 SD)	0.76 (0.71–0.82)	<0.001	0.77 (0.70–0.84)	<0.001	0.80 (0.73–0.87)	<0.001
Perceived organizational support (per 1 SD)	0.90 (0.83–0.97)	0.006	1.02 (0.94–1.10)	0.614	1.07 (0.99–1.17)	0.105

Modified Poisson regression with robust sandwich variance (22) was used to estimate prevalence ratios (PR). Model 1: adjusted for age, professional rank, department, employment status, night shifts, biosafety training, knowledge of reporting procedure, safety climate, and perceived organizational support (10 df). Model 2: Model 1 + psychological safety (11 df). Sex was excluded from adjusted models due to the small male sample (~5%). Variables not retained in adjusted models (marked with an em dash) were tested in unadjusted analysis only. aPR = adjusted prevalence ratio; CI = confidence interval; ref = reference category; SD = standard deviation. Values of p < 0.05 are shown in bold in the manuscript version.

**Figure 2:**
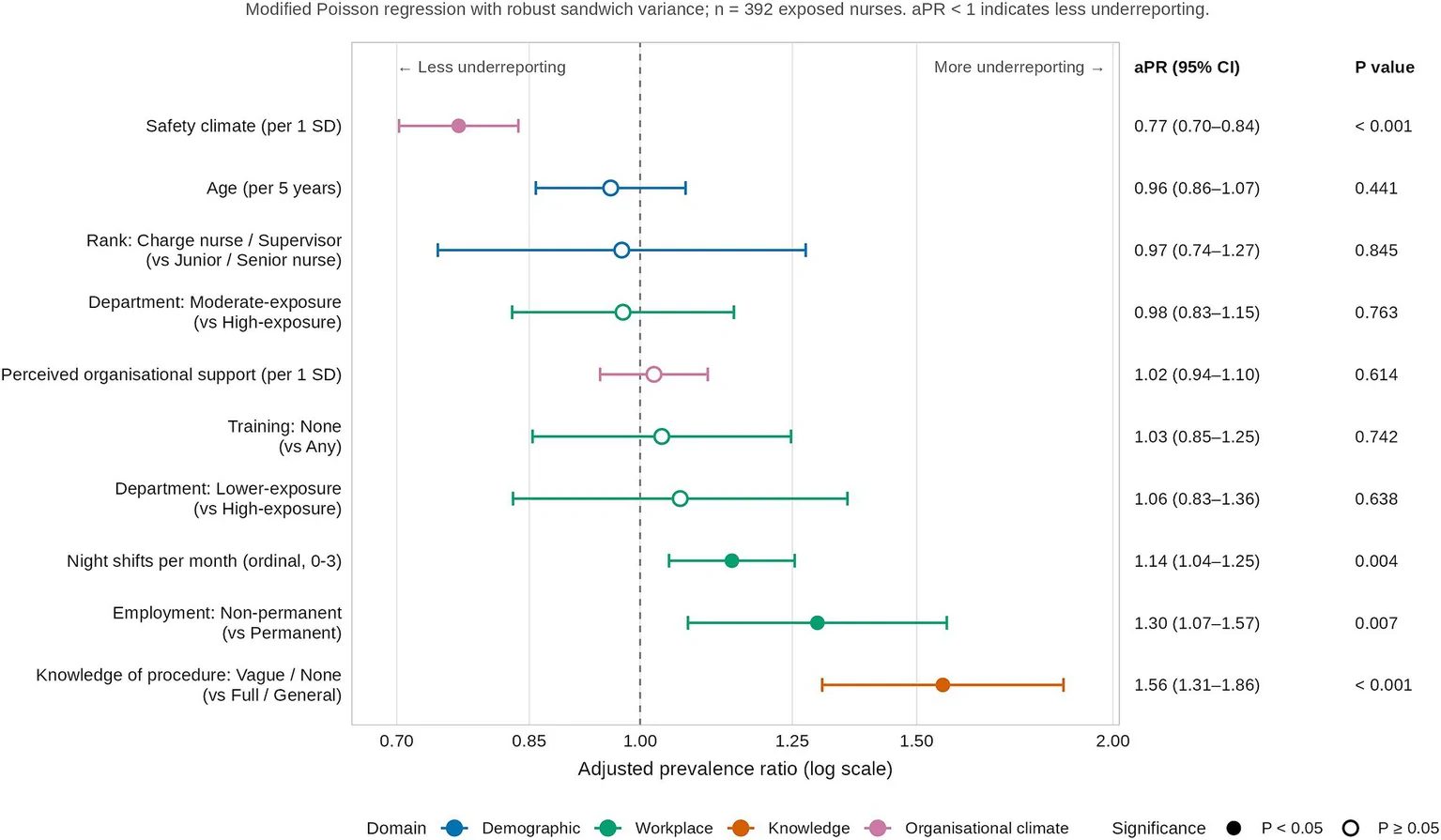
Adjusted prevalence ratios for underreporting of blood and body fluid exposures (Model 1). Modified Poisson regression with robust (HC0 sandwich) variance among exposed nurses; psychological safety was not included in Model 1; therefore, the safety-climate coefficient represents the association estimated without conditioning on psychological safety. Scale scores are per 1 SD, age is per 5 years, and night shifts are represented as an ordinal score of 0–3. Symbols are point estimates, and lines are 95% CIs on a logarithmic scale (filled, *p* < 0.05; open, *p* ≥ 0.05); color denotes covariate domain. aPR, adjusted prevalence ratio; CI, confidence interval; SD, standard deviation.

Four covariates retained independent associations in Model 1: each standard-deviation increase in safety climate was associated with a 23% lower prevalence (aPR 0.77, 0.70–0.84; *p* < 0.001), whereas vague or absent procedural knowledge (1.56, 1.31–1.86; *p* < 0.001), non-permanent employment (1.30, 1.07–1.57; *p* = 0.007), and night-shift frequency (1.14 per level, 1.04–1.25; *p* = 0.004) were each associated with a higher prevalence. Age (0.96, 0.86–1.07), rank (0.97, 0.74–1.27), clinical area, biosafety training (1.03, 0.85–1.25), and perceived organizational support (1.02, 0.94–1.10) were not independently associated; the attenuation of the crude age and rank associations suggests that they largely reflected correlated employment, work-organization, and organizational-perception characteristics. In Model 2, psychological safety was independently associated with a lower prevalence of underreporting (aPR 0.87, 95% CI 0.79–0.95; *p* = 0.001), and the safety-climate estimate changed modestly from 0.77 to 0.80 (0.73–0.87), with other estimates essentially unchanged. This change indicates overlapping information between the two perception scales and was not interpreted as mediation.

The exposure-offset negative-binomial model provided results consistent with the primary analysis for safety climate (IRR 0.76), procedural knowledge (IRR 1.53), non-permanent employment (IRR 1.45), and night-shift frequency (IRR 1.16), with all 95% CIs excluding 1 (Supplementary Table S3). In the hurdle model, all four characteristics were associated with the binary component (safety climate OR 0.42, 95% CI 0.32–0.56; procedural knowledge OR 3.81, 2.17–6.67; non-permanent employment OR 2.16, 1.27–3.66; night-shift frequency OR 1.47, 1.09–1.98), whereas only safety climate was associated with the positive-count component among under-reporters (IRR 0.80, 0.66–0.96). Firth penalized logistic regression provided similar ORs (0.44, 3.64, 2.11, and 1.45), and a likelihood-ratio test did not indicate evidence of departure from linearity for the ordinal night-shift score (*χ*^2^ = 1.34, df = 2, *p* = 0.511). The prespecified five-factor model for the reasons inventory showed inadequate fit [CFI 0.853, TLI 0.793, RMSEA 0.144 (90% CI 0.128–0.161), SRMR 0.143], and one item had a very low standardized loading (0.09) (Supplementary Figure S1); reasons were therefore analyzed at the item level.

### 3.6 Reasons for not reporting and proposed facilitators

Among the 232 under-reporters, the most strongly endorsed individual items concerned fear of being blamed or judged careless (mean 3.12; 104, 44.8, 95% CI 38.6–51.3, rating the item very much or extremely), fear of an effect on appraisal, contract, or promotion (3.13; 98, 42.2%), and worry that colleagues or managers would find out (3.07; 92, 39.7%) (Table 5; Figure 3). These were followed by a reporting process that was too long or complicated (3.00; 37.9%) and being too busy to leave clinical duties (2.98; 36.2%), whereas believing the source patient was uninfected (2.62; 21.6%) and judging the risk negligible (2.55; 22.0%) had the lowest proportions of strong endorsement. Items are presented individually; the *a priori* groupings in Table 5, Figure 3 are descriptive labels and not validated subscales. When asked for the single most important reason, responses were dispersed: a reporting process that was too long or complicated was selected most often (32, 13.8%), followed by not knowing how or where to report and fear of being blamed (29 each, 12.5%). The most frequently endorsed facilitators addressed the perceived consequences of reporting: an explicit no-blame policy (137, 59.1, 95% CI 52.6–65.2), an anonymous or confidential reporting option (136, 58.6%), assurance that reporting would not affect appraisal (133, 57.3%), and feedback on the outcome of a report (128, 55.2%). Process-related measures were endorsed less often (shorter form 48.7%, ward cover 47.0%, 24-h availability 39.2%), and only 10 nurses (4.3%) indicated that none of the eight measures would change their behavior.

**Table 5:** Reasons for not reporting and facilitators that would improve reporting behavior.

Part A. Extent of each reason for not reporting				
Domain^^†^^	Reason	Mean (SD)	% Very much/extremely	Rank
Risk minimization	Judged the risk negligible	2.55 (1.18)	22.0 (17.1–27.7)	11
Risk minimization	Source patient believed uninfected	2.62 (1.14)	21.6 (16.7–27.3)	12
Time/workload pressure	Reporting process too long or complicated	3.00 (1.23)	37.9 (31.9–44.3)	4
Time/workload pressure	Too busy to leave clinical duties	2.98 (1.27)	36.2 (30.3–42.6)	5
Knowledge gap	Did not know it should be reported	2.71 (1.23)	29.3 (23.8–35.5)	7
Knowledge gap	Did not know how or where to report	2.75 (1.31)	28.4 (23.0–34.6)	8
Fear of negative consequences	Feared being blamed or judged careless	3.12 (1.39)	44.8 (38.6–51.3)	1
Fear of negative consequences	Feared effect on appraisal, contract, or promotion	3.13 (1.33)	42.2 (36.1–48.7)	2
Fear of negative consequences	Worried colleagues or managers would find out	3.07 (1.28)	39.7 (33.6–46.1)	3
Futility and embarrassment	Embarrassed to admit the mistake	2.88 (1.18)	31.5 (25.8–37.7)	6
Futility and embarrassment	Believed reporting would change nothing	2.59 (1.33)	26.7 (21.4–32.8)	9
Futility and embarrassment	Previous report met an unhelpful response	2.61 (1.22)	24.1 (19.1–30.0)	10

Part B. Single most important reason (Q33)			
Reason	*n*	%	Rank
Reporting process too long or complicated	32	13.8	1
Did not know how or where to report	29	12.5	2
Feared being blamed or judged careless	29	12.5	3
Feared effect on appraisal, contract, or promotion	23	9.9	4
Worried colleagues or managers would find out	23	9.9	5
Too busy to leave clinical duties	18	7.8	6
Judged the risk negligible	16	6.9	7
Believed reporting would change nothing	15	6.5	8
Previous report met an unhelpful response	14	6.0	9
Did not know it should be reported	13	5.6	10
Embarrassed to admit the mistake	11	4.7	11
Source patient believed uninfected	9	3.9	12
Not applicable	0	0.0	13

Part C. Facilitators that would encourage reporting		
Facilitator	*n* (%)	95% CI
Explicit no-blame policy from management	137 (59.1)	52.6–65.2
Anonymous or confidential reporting option	136 (58.6)	52.2–64.8
Assurance that reporting will not affect appraisal	133 (57.3)	50.9–63.5
Clear feedback on what happened after a report	128 (55.2)	48.7–61.4
Shorter, simpler reporting form	113 (48.7)	42.3–55.1
Ward cover arranged to allow reporting	109 (47.0)	40.7–53.4
Reporting available 24 h a day	91 (39.2)	33.2–45.6
None of these would change behavior	10 (4.3)	2.4–7.8

Part A: Each reason was rated on a 5-point scale (1 = not at all, 5 = extremely). Mean (SD) and the proportion rating very much or extremely are shown. Items are ranked by the proportion rating very much or extremely. ^†^Groupings are a priori descriptive labels and not validated subscales; the five-factor structure was not supported. Part B: Nurses selected the single most important reason. Part C: Nurses endorsed facilitators that would change their reporting behavior. Wilson 95% confidence intervals are shown. PPE, personal protective equipment; PEP, post-exposure prophylaxis.

**Figure 3:**
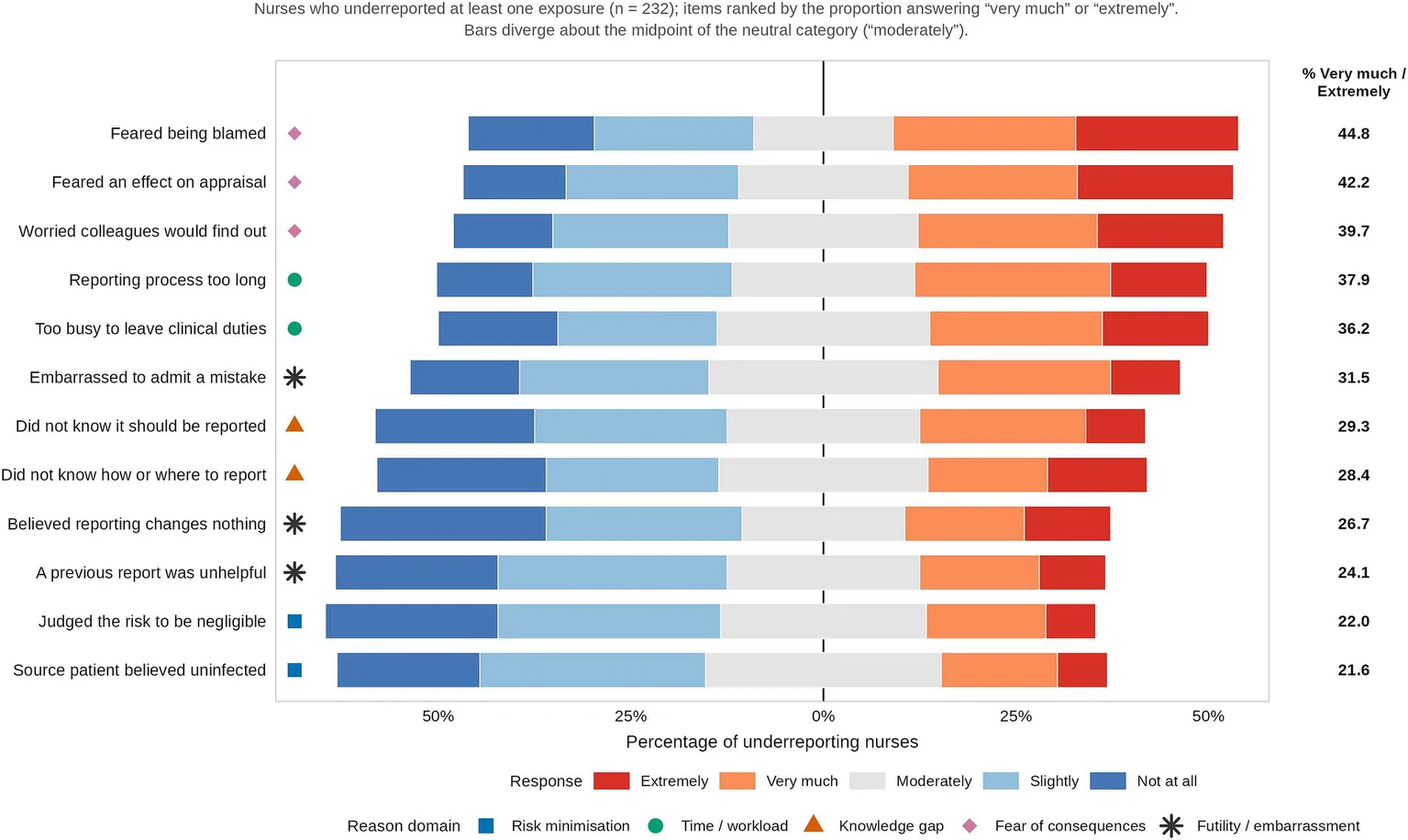
Reasons for not formally reporting a blood or body fluid exposure. Twelve reasons were rated on a 5-point intensity scale by nurses who underreported at least one exposure, with bars diverging from the midpoint of the neutral category and segment widths expressed as percentages of that cohort. Items are ordered by the proportion of nurses answering “very much” or “extremely,” shown in the right-hand column; symbols denote *a priori* descriptive groupings, which are not validated subscales.

## 4 Discussion

Nearly half of the nurses in this tertiary infectious disease hospital (45.0%) experienced a blood or body fluid exposure during the preceding 12 months, and 59.2% of exposed nurses failed to report at least one exposure. Across aggregate exposure counts, 373 of 747 exposures (49.9%) were not formally reported, indicating that, based on respondent reports, approximately half of exposures would not have been represented in formal reporting records. Of the 392 exposed nurses, 299 (76.3%) reported either all or none of their exposures, a pattern partly expected because most contributed only one or two exposures. After multivariable adjustment, underreporting was associated predominantly with organizational and procedural factors, including safety climate, psychological safety, employment security, night-shift frequency, and knowledge of the reporting pathway. In contrast, recent biosafety training, perceived organizational support, age, and professional rank were not independently associated with underreporting. Taken together, the associations, reported barriers, and preferred facilitators are consistent with the interpretation that anticipated personal consequences of reporting and the perceived credibility of institutional safeguards are relevant to reporting decisions.

The person-level underreporting prevalence of 59.2% closely approximates the 59.9% (95% CI 52.0–67.7) pooled across 41 studies comprising 19,635 healthcare workers in the largest meta-analysis of unreported needlestick injuries (6). It is also consistent with the pooled reporting rate of 42% (33–52%) across 21 Iranian nursing studies, corresponding to approximately 58% non-reporting (27). In contrast, the present estimate substantially exceeds the 18.4% overall non-reporting prevalence, including 10.2% among nurses, reported at a Saudi tertiary hospital (28) and the 28.5% observed among 6,464 healthcare workers in Gansu Province, China (8).

This heterogeneity may reflect differences in ascertainment as much as genuine institutional variation. Both lower estimates relied on a single global question concerning “the injury,” whereas this study separately enumerated percutaneous and mucocutaneous exposures and the number of exposures formally reported. This approach is particularly relevant because mucocutaneous exposures, which are often less consistently reported, accounted for 289 of the 747 events and are excluded from needlestick-only instruments. Furthermore, the questionnaire was anonymous and explicitly separated from staff appraisal, thereby reducing the likelihood that the survey itself reproduced the perceived consequences of the institutional reporting system. The potential magnitude of ascertainment bias is illustrated by the Cochrane review of educational interventions, in which registry-derived sharps injury rates ranged from 43 to 203 per 1,000 workers annually, whereas questionnaire-derived estimates ranged from 1,800 to 7,000 per 1,000 workers (29). This discrepancy underscores the substantial under-ascertainment that can occur when occupational injuries are estimated solely from routine institutional records.

The present findings are also consistent with the broader occupational literature, in which underreporting of work-related injuries ranges from 20 to 91% across sectors (30), and even non-infectious occupational conditions, such as work-related low back pain, are systematically withheld by registered nurses (31). Thus, the magnitude of underreporting observed here is not exceptional. More striking is its persistence in a workforce in which 88.6% had completed hepatitis B vaccination, and 85.4% had received recent biosafety training, suggesting that high coverage of preventive measures does not necessarily translate into complete reporting (48, 49).

Risk minimization was among the least endorsed, rather than the dominant, explanations for non-reporting. Previous studies have generally emphasized low perceived risk: the meta-analysis of unreported needlestick injuries identified lack of concern about the injury as the most frequently reported reason for non-reporting (6), whereas the Gansu study identified failure to perceive the source patient as infectious among the principal explanations (8). The present findings showed the opposite pattern. Among 12 potential reasons, believing that the source patient was uninfected (mean 2.62; 21.6% strong endorsement) and considering the exposure negligible (mean 2.55; 22.0%) ranked 12th and 11th, respectively. By contrast, concern that reporting could affect appraisal, contract renewal, or promotion (mean 3.13; 42.2%), fear of being blamed or perceived as careless (mean 3.12; 44.8%), and concern that colleagues or managers would become aware of the event (mean 3.07; 39.7%) were the most strongly endorsed reasons.

This divergence is plausible in an infectious disease hospital, where the possibility of a clinically important source-patient infection is difficult to discount. For the most recent exposure, 23.0% of nurses reported a source known to be positive for HIV, hepatitis B, or hepatitis C, and a further 52.6% reported a source whose infection status was unknown or could not be established. Under these conditions, non-reporting may depend less on minimization of biological risk and more on anticipated social or occupational consequences. This interpretation is consistent with qualitative evidence identifying fear of blame as an important determinant of reporting behavior in high-acuity healthcare settings (12, 13). Accordingly, interventions focused exclusively on increasing awareness of bloodborne-pathogen risk may be less effective where such risk is already salient and the principal deterrents are organizational factors.

Recent biosafety training was also not independently associated with underreporting, in contrast to the previous studies in which training frequency was among the strongest correlates of reporting (28, 32). In the present cohort, biosafety training during the preceding 12 months was unrelated to underreporting in either crude or adjusted analyses (aPR 1.03, 0.85–1.25; *p* = 0.742) and did not distinguish exposed from unexposed nurses (*p* = 0.778). Several factors may explain this finding. First, recent training was highly prevalent (85.4%), leaving little variation for detecting between-group differences; moreover, a binary measure of attendance does not capture training frequency, content, recency, or competence. Second, attendance did not consistently translate into procedural knowledge: 41.6% of nurses could describe the institutional reporting procedure only vaguely, whereas procedural knowledge showed a strong independent association with underreporting (aPR 1.56, 1.31–1.86). A similar pattern emerged in the Saudi study, where non-reporting concentrated among healthcare workers unaware of the reporting protocol (28). Third, evidence on interventions remains limited. The Cochrane review identified only seven eligible studies, most of which were non-randomized, resulting in low-certainty and imprecise estimates (29). In contrast, the Chinese study demonstrating improvements in knowledge, behavior, and injury incidence evaluated an intensive, protocol-specific intervention among student nurses (32), rather than routine mandatory training. These findings suggest that procedural competence may be a more informative training endpoint than attendance alone (50).

Perceived organizational support likewise did not remain independently associated after adjustment. Although greater perceived organizational support was associated with less underreporting in crude analysis (PR 0.90, 0.83–0.97), this association attenuated after adjustment (aPR 1.02 in Model 1 and 1.07 in Model 2), whereas safety climate and psychological safety remained independently associated. This distinction may reflect differences in construct specificity. Perceived organizational support represents a broad belief that the employer values and supports staff, whereas safety climate and psychological safety more directly capture whether disclosing an incident is expected to result in constructive rather than punitive consequences. The moderate correlations among these scales (0.41–0.44) further suggest that part of the crude association with generalized organizational support may reflect shared variance with more reporting-specific constructs. Other nursing research has reported that psychological safety mediates associations between leadership and speaking up or error reporting (33), whereas willingness to raise safety concerns may operate through commitment-based safety management rather than generalized organizational perceptions (34). Thus, improving general staff support may be insufficient if the perceived consequences of incident disclosure remain unchanged.

Age and professional seniority were also not independently associated with underreporting. Although underreporting decreased substantially with age in crude analysis (PR 0.83 per 5 years, 0.76–0.90) and varied by professional rank, both associations disappeared after multivariable adjustment (age aPR 0.96, 0.86–1.07; rank aPR 0.97, 0.74–1.27). This finding is consistent with meta-analytic evidence showing no association of mean age or years of experience with either injury prevalence or reporting rates across Iranian nursing studies (27). However, it contrasts with findings from Gansu, where more experienced healthcare workers both sustained and did not report more injuries (8). In the present cohort, younger and more junior nurses were more frequently non-permanent employees, worked more night shifts, carried heavier workloads, and reported a weaker safety climate. Adjusting for these conditions substantially attenuated the demographic associations, suggesting that age and seniority may primarily reflect underlying employment and work-organization characteristics. A comparable pattern has been observed for temporary employment and occupational injury, in which a 2.5-fold to 3-fold crude excess was attenuated after adjustment for duration of employment (35). These findings suggest that preventive strategies may benefit from prioritizing modifiable working conditions rather than demographic categories alone.

Safety climate showed the strongest independent association with underreporting (aPR 0.77 per SD, 95% CI 0.70–0.84). Because two safety-climate items referred directly to blame and institutional responses to incident reports, part of this association may reflect conceptual overlap with the reporting outcome. However, perceived organizational support, measured in the same questionnaire but not specifically related to incident reporting, was not associated with underreporting after adjustment (aPR 1.02, 0.94–1.10), which provides some evidence against a purely generalized same-source response tendency. Residual criterion contamination and common-method variance nevertheless cannot be excluded. Psychological safety was also independently associated with underreporting (aPR 0.87, 0.79–0.95), and its inclusion changed the safety-climate estimate only modestly (from 0.77 to 0.80). This pattern suggests that the two scales capture partly overlapping perceptions of how disclosure is received, but it does not establish a causal pathway, direction of influence, or mediation. The persistence of both associations is consistent with systematic review evidence showing that self-preservation motives inhibit error reporting, whereas favorable perceptions of organizational and leadership responses promote disclosure (36).

Non-permanent employment was independently associated with a 30% higher prevalence of underreporting (aPR 1.30, 1.07–1.57), with consistent associations in the count-based and hurdle sensitivity analyses. This finding is consistent with register-based evidence indicating that occupational injuries are approximately 50% more likely to be underreported among precariously employed workers (37) and with systematic review evidence linking precarious employment with occupational injury more broadly (35). Because 50.6% of nurses in the present workforce held non-permanent contracts, employment insecurity may have substantial implications for the completeness of institutional exposure surveillance. Importantly, concern about contract renewal, appraisal, and promotion was also among the most frequently endorsed explanations for not reporting exposures.

Night-shift frequency, modeled as an ordinal score, was independently associated with underreporting (aPR 1.14 per level, 1.04–1.25; 1.16 in Model 2). Shift work and sleep disturbance are established determinants of occupational injury risk (38, 39); the present findings suggest that shift organization may also be associated with whether an exposure is subsequently reported. One plausible mechanism is reduced access to occupational health services: 41.1% of most recent exposures occurred during the second half of a shift and 15.1% during unscheduled overtime, periods in which staffing may be reduced and leaving the clinical unit may be more difficult. Nevertheless, only 39.2% of under-reporters identified 24-h reporting availability as a facilitator, suggesting that improved after-hours access, although potentially important, is unlikely to address the broader organizational barriers to reporting (6, 12).

The concentration of exposed nurses in the all-reported (40.8%) and none-reported (35.5%) categories requires cautious interpretation. Nurses with a single exposure could only report all or none of their exposures, and nurses with more exposures had more opportunities to be classified as under-reporters even if the probability of reporting each exposure was unchanged. The observed distribution therefore cannot establish a behavioral threshold. The exposure-offset negative-binomial model, which related each nurse’s unreported exposures to total exposures, yielded associations consistent with the nurse-level analysis, suggesting that the principal correlates were not explained solely by differences in exposure opportunity, although this model does not formally constrain unreported counts to their total. Among nurses whose most recent exposure was reported, reporting intervals (*p* = 0.759), reporting within 24 h (88.1% *vs*. 91.4%; *p* = 0.771), and completion of advised prophylaxis (61.6% *vs*. 71.0%; *p* = 0.470) were similar, although these comparisons included few under-reporters. Taken together, these findings suggest that initiating a formal report may be an important decision point, and interventions to reduce barriers to initial reporting warrant evaluation alongside efforts to support downstream follow-up (7, 12–14, 26).

Because post-exposure services at this institution are accessed through the reporting pathway, differences in subsequent care are structurally linked to reporting status and should not be interpreted as causal effects of reporting itself. Their magnitude nevertheless indicates the potential clinical importance of underreporting. Among 232 under-reporters, 112 (48.3%) reported receiving none of five basic post-exposure services, compared with 1 of 160 complete reporters (0.6%). Baseline serological testing was performed in 12.1% *versus* 83.8%, hepatitis B immunoglobulin or booster vaccination was received by 3.9% *versus* 30.6%, and HIV PEP by 2.2% *versus* 23.8%, respectively.

These differences may be clinically consequential because HIV PEP is time-sensitive and hepatitis B PEP is most effective when administered within 24 h (4, 5). Moreover, 23.0% of nurses reported that their most recent exposure involved a source known to be positive for a bloodborne pathogen. However, reporting behavior and service receipt were both measured at the nurse level, and the questionnaire did not link services to individual exposures. An underreporting nurse may have received testing, vaccination, or prophylaxis after a different exposure that was formally reported, and clinical indications for each service could not be determined. The findings therefore show a strong association between an underreporting pattern and lower nurse-level receipt of post-exposure care, but they do not establish that each of the 373 unreported exposures resulted in a missed, clinically indicated service. Underreporting may nonetheless compromise both individual post-exposure protection and the institution’s capacity to characterize occupational infection risk.

The facilitators most frequently endorsed by nurses corresponded to the correlates identified in the multivariable analyses and to the most strongly endorsed reasons for not reporting. An explicit no-blame policy (59.1%), anonymous or confidential reporting (58.6%), assurance that reporting would not influence appraisal (57.3%), and feedback regarding report outcomes (55.2%) were endorsed more frequently than shorter reporting forms (48.7%), ward cover during reporting (47.0%), or 24-h reporting availability (39.2%). Only 4.3% indicated that no intervention would alter their reporting behavior, suggesting that organizational improvement may be feasible. Fear of punitive consequences and concerns about confidentiality are consistently identified barriers in contemporary incident-reporting research (40, 41), while implementation of an anonymous and confidential electronic reporting system in another tertiary hospital was associated with a 33.6% increase in incident reports (*p* < 0.0001) (42). Given that evidence for discrete interventions to improve psychological safety remains mixed and based on relatively few objective outcomes (43), the present findings support evaluating credible institutional safeguards, including explicit non-punitive reporting policies, formal separation of exposure reporting from appraisal and contract renewal, confidentiality protections, and closed-loop feedback to reporters.

Two additional employer-level priorities emerge from the exposure data. First, procedural competence should become an explicit endpoint of biosafety education. Despite 85.4% recent training coverage, 41.6% of nurses could not clearly describe the reporting pathway, and procedural knowledge was independently associated with underreporting. Training effectiveness should therefore be assessed through demonstrated knowledge of reporting procedures rather than attendance alone. Second, deficits in safety equipment may indicate a broader working environment in which both exposure prevention and reporting are compromised. Under-reporters rated unavailable safety-engineered devices, full or inaccessible sharps containers, and unavailable or poorly fitting personal protective equipment as more important contributors than did complete reporters, with all corresponding graded comparisons reaching *p* ≤ 0.011. In contrast, rushing, fatigue, and failure to follow procedures did not distinguish the groups. This pattern suggests that differences in perceived working conditions may be more salient than explanations centered on individual carelessness. Safety-engineered devices reduce needlestick injuries, with moderate-quality evidence indicating pooled RRs of 0.28 (0.13–0.59) for intravenous devices and 0.57 (0.38–0.84) for phlebotomy devices (14). However, Cochrane evidence is less definitive and indicates that actively engaged safety systems may increase blood splashes (RR 1.6, 1.08–2.36) (44). These findings favor passive safety designs where feasible and reinforce the importance of appropriate eye protection, particularly because the eyes or conjunctiva accounted for 40.9% of mucocutaneous exposures.

This study has several strengths. Percutaneous and mucocutaneous exposures and the number formally reported were elicited separately rather than through a single global item, allowing underreporting to be estimated both at the nurse level and as a proportion of aggregate exposure counts. Count-based classifications were consistent with a separately worded categorical reporting item, although this concordance represents internal consistency rather than independent validation. The single-site design held the formal reporting policy, PEP pathway, and occupational health service constant, and the census approach with a 78.6% response rate reduced, but did not eliminate, the potential for selection bias.

Several limitations should be considered. First, the cross-sectional design and concurrent self-reporting of exposures, reporting behavior, organizational perceptions, and service receipt preclude temporal or causal inference; reverse associations and common-method variance are plausible. Two safety-climate items referred to institutional responses to incident reporting and may overlap conceptually with the outcome. Second, the 12-month recall period may have produced differential recall, particularly of minor or earlier exposures, and exposure counts could not be verified against institutional records. Third, the nurse-level definition of at least one unreported exposure is structurally influenced by exposure frequency, and night-shift frequency and non-permanent employment were more common among exposed than unexposed nurses. The principal associations may therefore partly reflect exposure opportunity. The exposure-offset analysis produced consistent estimates but does not formally constrain unreported counts to the total experienced, and the concentration of respondents in the all-reported and none-reported categories cannot establish a behavioral threshold. Fourth, the 747 exposures were aggregate respondent-level counts rather than individually characterized records. Detailed characteristics concerned the most recent exposure, whereas post-exposure services were recorded as receipt at least once after any exposure; therefore, source status, clinical risk, prophylaxis indication, and management cannot be assigned to the 373 unreported counts. Fifth, the translated organizational scales require further external validation, and the reasons inventory did not support its prespecified factor structure; consequently, reasons were interpreted only at the item level. Sixth, despite *a priori* covariate selection, residual confounding, overadjustment, and collinearity among concurrently measured perceptual scales remain possible. Seventh, individual-level information was unavailable for the 238 non-respondents, whose participation may have differed by exposure history, willingness to disclose, employment security, workload, or organizational perceptions; the direction and magnitude of non-response bias cannot be determined. Finally, organizational measures represented individual perceptions within a single hospital, precluding estimation of hospital-level effects or causal effects of policy change and limiting generalizability to other institutions. The specialized setting may also have shaped risk perception and reporting motivations. Because 75.6% of most recent exposures involved a source that was known to be positive or of unknown status, bloodborne risk may have been highly salient, which may explain why risk minimization ranked low among reasons for non-reporting, in contrast with studies from general healthcare settings. The absolute prevalence of underreporting and the ranking of reasons may therefore differ in general hospitals, whereas the associations with safety climate, procedural knowledge, employment security, and night-shift work may be more transferable, pending multicenter confirmation.

## 5 Conclusion

Underreporting of blood and body fluid exposures was common among nurses in this tertiary infectious disease hospital: 59.2% of exposed nurses failed to report at least one exposure, and 49.9% of the 747 aggregate exposure counts were unreported. Individual perceptions of a stronger safety climate were associated with less underreporting, whereas vague or absent procedural knowledge, non-permanent employment, and more frequent night shifts were associated with greater underreporting. These findings identify potentially actionable conditions but do not demonstrate that institutional policy changes would cause the estimated differences. Similarly, lower nurse-level receipt of post-exposure services cannot be linked to specific unreported exposures or interpreted as evidence that clinically indicated prophylaxis was omitted. Prospective multicenter studies using individually characterized exposure records are required to evaluate causal mechanisms and intervention effectiveness.

## Data Availability

The raw data supporting the conclusions of this article will be made available by the authors, without undue reservation.

## Supplementary material

SUPPLEMENTARY FIGURE S1 Confirmatory factor analysis of the 12-item reasons-for-not-reporting scale. Robust diagonally weighted least-squares (WLSMV) estimation of the prespecified five-factor structure was performed among nurses who underreported at least one exposure, with items treated as ordered categorical and loadings constrained to be equal within the three two-item factors for identification. Ellipses are latent factors, rectangles are observed items, solid arrows are standardized loadings, and dashed curves represent inter-factor correlations; fit indices are shown in the panel, and the loading in red indicates a probable cross-loading. CFI, comparative fit index; RMSEA, root mean square error of approximation; SRMR, standardized root mean square residual; TLI, Tucker–Lewis index.
