# Supplementary material for: Underreporting of blood and body fluid exposures among nurses: prevalence, reporting cascade, and modifiable organizational correlates
Source: Front Public Health. 2026 Oct 7;14:1989101. doi: 10.3389/fpubh.2026.1989101 (PMC13683579; doi:10.3389/fpubh.2026.1989101)
Supplement: Supplementary file 1 [file Table_1.DOCX]

**Table S1. Full 45-item survey instrument with response options and routing rules**

| **Item code** | **Section** | **Question** | **Response type** | **Response options** | **Administered to** |
| --- | --- | --- | --- | --- | --- |
| ID | — | Anonymous respondent identifier | String | N0001 to N0872 | All 872 respondents |
| Q1_age | A | Age in completed years | Continuous | Whole years, 21 to 57 | All 872 respondents |
| Q2_sex | A | Sex | Nominal | Female; Male; Prefer not to say | All 872 respondents |
| Q3_education | A | Highest nursing qualification | Ordinal | Diploma in nursing; Bachelor's degree (BSN or equivalent); Master's degree or higher; Other | All 872 respondents |
| Q4_rank | A | Professional rank or title | Ordinal | Staff nurse / junior nurse; Senior staff nurse; Charge nurse / ward in-charge; Nursing supervisor or above | All 872 respondents |
| Q5_years_total | A | Total years worked as a nurse | Ordinal | Less than 2 years; 2 to 5 years; 6 to 10 years; More than 10 years | All 872 respondents |
| Q6_years_hospital | A | Years worked at this hospital | Ordinal | Less than 2 years; 2 to 5 years; 6 to 10 years; More than 10 years | All 872 respondents |
| Q7_employment | A | Employment status | Nominal | Permanent / regular staff; Contract staff; Agency, deputed, or temporary staff; Intern or nurse in training | All 872 respondents |
| Q8_department | A | Main clinical area | Nominal | General infectious disease ward; Isolation or high-consequence pathogen unit; Intensive care unit; Emergency department; Outpatient or fever clinic; Operating room or procedure suite; Dialysis unit; Other | All 872 respondents |
| Q9_nightshifts | A | Night shifts per month | Ordinal | No night shifts; 1 to 4 per month; 5 to 8 per month; More than 8 per month | All 872 respondents |
| Q10_patientload | A | Patients directly responsible for per shift | Ordinal | Fewer than 5; 5 to 9; 10 to 14; 15 or more | All 872 respondents |
| Q11_HBV_vaccine | B | Hepatitis B vaccination status | Nominal | Completed the full course (3 or more doses); Received 1 or 2 doses only; Never vaccinated; Not sure | All 872 respondents |
| Q12_antiHBs | B | Anti-HBs level ever checked | Nominal | Yes, and I was protected; Yes, and I was not protected; No; Not sure | All 872 respondents |
| Q13_training | B | Biosafety training in the past 12 months | Ordinal | Yes, more than once; Yes, once; No | All 872 respondents |
| Q14_know_procedure | B | Knowledge of the official reporting procedure | Ordinal | I know the full procedure and could follow it without help; I know the general steps but would need to check some details; I have only a vague idea of the procedure; I do not know the procedure at all | All 872 respondents |
| Q15_know_PEP_access | B | Knows PEP location and after-hours access | Ordinal | Yes, both; I know the location but not the after-hours access; I know neither | All 872 respondents |
| Q16_percutaneous_n | C | Number of percutaneous injuries, past 12 months | Ordinal | No percutaneous injuries; One injury; Two injuries; Three injuries; Four injuries; Five or more injuries | All 872 respondents |
| Q17_device | C | Device in most recent percutaneous injury | Nominal | Hollow-bore hypodermic needle; Intravenous cannula or stylet; Blood collection needle or vacutainer; Suture needle; Lancet; Scalpel or blade; Broken glass or ampoule; Other | Only if Q16 is not "None"; others read "Not applicable" |
| Q18_activity | C | Activity at time of most recent percutaneous injury | Nominal | Giving an injection or inserting a cannula; Drawing blood; Recapping a needle; Disposing of a sharp into a container; Handling or transporting used equipment or linen; Cleaning up after a procedure; Assisting another staff member during a procedure; Other | Only if Q16 is not "None"; others read "Not applicable" |
| Q19_gloves | C | Gloves worn at time of injury | Nominal | Yes, double gloves; Yes, single gloves; No | Only if Q16 is not "None"; others read "Not applicable" |
| Q20_mucocutaneous_n | C | Number of mucocutaneous exposures, past 12 months | Ordinal | No mucocutaneous exposures; One exposure; Two exposures; Three exposures; Four exposures; Five or more exposures | All 872 respondents |
| Q21_site | C | Body site of most recent mucocutaneous exposure | Nominal | Eyes or conjunctiva; Nose or mouth; Broken or damaged skin; More than one site | Only if Q20 is not "None"; others read "Not applicable" |
| Q22_source_status | C | Source patient infectious status at the time | Nominal | Yes, known to be positive for a bloodborne pathogen (HIV, HBV, or HCV); Yes, known to be negative; Not known at the time; The source patient could not be identified | Only nurses with an exposure; others read "Not applicable" |
| Q23_shift_timing | C | Point in the shift when the exposure occurred | Nominal | First half of the shift; Second half of the shift; During overtime beyond my scheduled hours; Do not recall | Only nurses with an exposure; others read "Not applicable" |
| Q24a | C | Contributing factor: rushing because of heavy workload | Ordinal | Not at all; Slightly; Moderately; Very much; Extremely | Only nurses with an exposure; others read "Not applicable" |
| Q24b | C | Contributing factor: fatigue or extended shift | Ordinal | Not at all; Slightly; Moderately; Very much; Extremely | Only nurses with an exposure; others read "Not applicable" |
| Q24c | C | Contributing factor: patient moved unexpectedly or was uncooperative | Ordinal | Not at all; Slightly; Moderately; Very much; Extremely | Only nurses with an exposure; others read "Not applicable" |
| Q24d | C | Contributing factor: safety-engineered devices unavailable | Ordinal | Not at all; Slightly; Moderately; Very much; Extremely | Only nurses with an exposure; others read "Not applicable" |
| Q24e | C | Contributing factor: sharps containers full, absent, or out of reach | Ordinal | Not at all; Slightly; Moderately; Very much; Extremely | Only nurses with an exposure; others read "Not applicable" |
| Q24f | C | Contributing factor: PPE unavailable or ill-fitting | Ordinal | Not at all; Slightly; Moderately; Very much; Extremely | Only nurses with an exposure; others read "Not applicable" |
| Q24g | C | Contributing factor: did not follow the correct procedure | Ordinal | Not at all; Slightly; Moderately; Very much; Extremely | Only nurses with an exposure; others read "Not applicable" |
| Q25_reporting_category | D | Proportion of exposures formally reported | Ordinal | All of them; Most but not all; Some of them; None of them | Only nurses with an exposure; others read "Not applicable" |
| Q26a_total_exposures | D | Total exposures experienced, past 12 months | Count | Whole number | Only nurses with an exposure; others read "Not applicable" |
| Q26b_total_reported | D | Total exposures formally reported | Count | Whole number, never exceeds Q26a | Only nurses with an exposure; others read "Not applicable" |
| Q27_recent_reported | D | Most recent exposure formally reported | Nominal | Yes, I reported it myself; Yes, someone else reported it on my behalf; No, it was never formally reported | Only nurses with an exposure; others read "Not applicable" |
| Q28_time_to_report | D | Time from exposure to report | Ordinal | Within 1 hour; Within 2 to 8 hours; Within 9 to 24 hours; More than 24 hours later; Not applicable, it was not reported | Only nurses with an exposure; others read "Not applicable" |
| Q29a | D | Received after exposure: immediate wound care or irrigation advice | Binary | Not selected; Selected | Only nurses with an exposure; others read "Not applicable" |
| Q29b | D | Received after exposure: baseline blood testing | Binary | Not selected; Selected | Only nurses with an exposure; others read "Not applicable" |
| Q29c | D | Received after exposure: hepatitis B immunoglobulin or booster | Binary | Not selected; Selected | Only nurses with an exposure; others read "Not applicable" |
| Q29d | D | Received after exposure: HIV post-exposure prophylaxis | Binary | Not selected; Selected | Only nurses with an exposure; others read "Not applicable" |
| Q29e | D | Received after exposure: counseling or psychological support | Binary | Not selected; Selected | Only nurses with an exposure; others read "Not applicable" |
| Q29f | D | Received after exposure: none of the above | Binary | Not selected; Selected | Only nurses with an exposure; others read "Not applicable" |
| Q30_PEP_completion | D | Completed the recommended PEP and follow-up | Nominal | Yes, completed in full; Started but did not complete; Was advised but never started; Not applicable | Only nurses with an exposure; others read "Not applicable" |
| Q31_noncompletion_reason | D | Main reason follow-up was not completed | Nominal | Side effects of the medication; Too busy or could not get time away from the ward; Believed the risk was too low to justify it; Follow-up appointments were inconvenient or poorly scheduled; Concerned about others finding out; Not applicable; Other | Only nurses with an exposure; others read "Not applicable" |
| Q32a | E | Reason for not reporting: judged the risk negligible | Ordinal | Not at all; Slightly; Moderately; Very much; Extremely | Only nurses who failed to report at least one exposure; others read "Not applicable" |
| Q32b | E | Reason for not reporting: source patient believed uninfected | Ordinal | Not at all; Slightly; Moderately; Very much; Extremely | Only nurses who failed to report at least one exposure; others read "Not applicable" |
| Q32c | E | Reason for not reporting: too busy to leave clinical duties | Ordinal | Not at all; Slightly; Moderately; Very much; Extremely | Only nurses who failed to report at least one exposure; others read "Not applicable" |
| Q32d | E | Reason for not reporting: reporting process too long or complicated | Ordinal | Not at all; Slightly; Moderately; Very much; Extremely | Only nurses who failed to report at least one exposure; others read "Not applicable" |
| Q32e | E | Reason for not reporting: did not know it should be reported | Ordinal | Not at all; Slightly; Moderately; Very much; Extremely | Only nurses who failed to report at least one exposure; others read "Not applicable" |
| Q32f | E | Reason for not reporting: did not know how or where to report | Ordinal | Not at all; Slightly; Moderately; Very much; Extremely | Only nurses who failed to report at least one exposure; others read "Not applicable" |
| Q32g | E | Reason for not reporting: feared being blamed or judged careless | Ordinal | Not at all; Slightly; Moderately; Very much; Extremely | Only nurses who failed to report at least one exposure; others read "Not applicable" |
| Q32h | E | Reason for not reporting: feared effect on appraisal, contract, promotion | Ordinal | Not at all; Slightly; Moderately; Very much; Extremely | Only nurses who failed to report at least one exposure; others read "Not applicable" |
| Q32i | E | Reason for not reporting: worried colleagues or managers would find out | Ordinal | Not at all; Slightly; Moderately; Very much; Extremely | Only nurses who failed to report at least one exposure; others read "Not applicable" |
| Q32j | E | Reason for not reporting: believed reporting would change nothing | Ordinal | Not at all; Slightly; Moderately; Very much; Extremely | Only nurses who failed to report at least one exposure; others read "Not applicable" |
| Q32k | E | Reason for not reporting: previous report met an unhelpful response | Ordinal | Not at all; Slightly; Moderately; Very much; Extremely | Only nurses who failed to report at least one exposure; others read "Not applicable" |
| Q32l | E | Reason for not reporting: embarrassed to admit the mistake | Ordinal | Not at all; Slightly; Moderately; Very much; Extremely | Only nurses who failed to report at least one exposure; others read "Not applicable" |
| Q33_main_reason | E | Single most important reason for not reporting | Nominal | Judged the risk negligible; Source patient believed uninfected; Too busy to leave clinical duties; Reporting process too long or complicated; Did not know it should be reported; Did not know how or where to report; Feared being blamed or judged careless; Feared effect on appraisal, contract, or promotion; Worried colleagues or managers would find out; Believed reporting would change nothing; Previous report met an unhelpful response; Embarrassed to admit the mistake | Only nurses who failed to report at least one exposure; others read "Not applicable" |
| Q34a | E | Would increase reporting: shorter, simpler reporting form | Binary | Not selected; Selected | Only nurses who failed to report at least one exposure; others read "Not applicable" |
| Q34b | E | Would increase reporting: anonymous or confidential reporting option | Binary | Not selected; Selected | Only nurses who failed to report at least one exposure; others read "Not applicable" |
| Q34c | E | Would increase reporting: explicit no-blame policy stated by management | Binary | Not selected; Selected | Only nurses who failed to report at least one exposure; others read "Not applicable" |
| Q34d | E | Would increase reporting: reporting available 24 hours a day | Binary | Not selected; Selected | Only nurses who failed to report at least one exposure; others read "Not applicable" |
| Q34e | E | Would increase reporting: ward cover arranged to allow reporting | Binary | Not selected; Selected | Only nurses who failed to report at least one exposure; others read "Not applicable" |
| Q34f | E | Would increase reporting: clear feedback on what happened after a report | Binary | Not selected; Selected | Only nurses who failed to report at least one exposure; others read "Not applicable" |
| Q34g | E | Would increase reporting: assurance that reporting will not affect appraisal | Binary | Not selected; Selected | Only nurses who failed to report at least one exposure; others read "Not applicable" |
| Q34h | E | Would increase reporting: none of these would change behavior | Binary | Not selected; Selected | Only nurses who failed to report at least one exposure; others read "Not applicable" |
| Q35a | F | Psychological safety: mistakes are often held against me (REVERSE-SCORED) | Ordinal | Strongly disagree; Disagree; Neutral; Agree; Strongly agree | All 872 respondents |
| Q35b | F | Psychological safety: team can raise problems and difficult issues | Ordinal | Strongly disagree; Disagree; Neutral; Agree; Strongly agree | All 872 respondents |
| Q35c | F | Psychological safety: people sometimes reject others for being different (REVERSE-SCORED) | Ordinal | Strongly disagree; Disagree; Neutral; Agree; Strongly agree | All 872 respondents |
| Q35d | F | Psychological safety: it is safe to take a risk on this team | Ordinal | Strongly disagree; Disagree; Neutral; Agree; Strongly agree | All 872 respondents |
| Q35e | F | Psychological safety: difficult to ask other team members for help (REVERSE-SCORED) | Ordinal | Strongly disagree; Disagree; Neutral; Agree; Strongly agree | All 872 respondents |
| Q35f | F | Psychological safety: no one would deliberately undermine my efforts | Ordinal | Strongly disagree; Disagree; Neutral; Agree; Strongly agree | All 872 respondents |
| Q35g | F | Psychological safety: my skills and talents are valued and used | Ordinal | Strongly disagree; Disagree; Neutral; Agree; Strongly agree | All 872 respondents |
| Q36a | F | Safety climate: management treats occupational safety as a genuine priority | Ordinal | Strongly disagree; Disagree; Neutral; Agree; Strongly agree | All 872 respondents |
| Q36b | F | Safety climate: staffing levels are adequate to work safely | Ordinal | Strongly disagree; Disagree; Neutral; Agree; Strongly agree | All 872 respondents |
| Q36c | F | Safety climate: safety-engineered sharps devices are consistently available | Ordinal | Strongly disagree; Disagree; Neutral; Agree; Strongly agree | All 872 respondents |
| Q36d | F | Safety climate: PPE is consistently available in my size | Ordinal | Strongly disagree; Disagree; Neutral; Agree; Strongly agree | All 872 respondents |
| Q36e | F | Safety climate: incident reports are used to improve practice, not to assign blame | Ordinal | Strongly disagree; Disagree; Neutral; Agree; Strongly agree | All 872 respondents |
| Q36f | F | Safety climate: staff who report incidents are supported rather than criticized | Ordinal | Strongly disagree; Disagree; Neutral; Agree; Strongly agree | All 872 respondents |
| Q37a | F | Organizational support: the hospital genuinely cares about my wellbeing | Ordinal | Strongly disagree; Disagree; Neutral; Agree; Strongly agree | All 872 respondents |
| Q37b | F | Organizational support: the hospital would help me if I had a problem at work | Ordinal | Strongly disagree; Disagree; Neutral; Agree; Strongly agree | All 872 respondents |
| Q37c | F | Organizational support: the hospital values my contribution | Ordinal | Strongly disagree; Disagree; Neutral; Agree; Strongly agree | All 872 respondents |
| Q37d | F | Organizational support: I could count on the hospital to support me after an occupational injury | Ordinal | Strongly disagree; Disagree; Neutral; Agree; Strongly agree | All 872 respondents |
| Q38_workload | G | Usual workload during a shift | Ordinal | Very light; Light; Moderate; Heavy; Very heavy | All 872 respondents |
| Q39_exhaustion | G | Frequency of working while physically exhausted | Ordinal | Never; Rarely; Sometimes; Often; Almost every shift | All 872 respondents |
| Q40_presenteeism | G | Frequency of working while unwell | Ordinal | Never; Once; 2 to 3 times; 4 to 5 times; More than 5 times | All 872 respondents |
| Q41_PPE_burden | G | Full PPE makes procedures harder to perform safely | Ordinal | Strongly disagree; Disagree; Neutral; Agree; Strongly agree | All 872 respondents |
| Q42_future_intention | G | Likelihood of reporting the next exposure | Ordinal | Very unlikely; Unlikely; Unsure; Likely; Very likely | All 872 respondents |
| Q43_perceived_risk | G | Perceived personal risk of bloodborne infection | Ordinal | Very low; Low; Moderate; High; Very high | All 872 respondents |
| Q44_anonymity_confidence | G | Confidence that responses remain anonymous | Ordinal | Not at all confident; Slightly confident; Moderately confident; Very confident; Completely confident | All 872 respondents |
| Q45_comment | G | Free-text comment | String | Open text; reads "No comment" where nothing was written | All 872 respondents |
| DV_exposed_any | Derived | Any exposure in the past 12 months | Binary | No; Yes | All 872 respondents |
| DV_n_exposures | Derived | Total exposures (Q16 + Q20) | Count | Whole number; 0 if unexposed | All 872 respondents |
| DV_n_reported | Derived | Total exposures formally reported | Count | Whole number | All 872 respondents |
| DV_n_unreported | Derived | Exposures not reported | Count | DV_n_exposures minus DV_n_reported | All 872 respondents |
| DV_underreported_any | Derived | PRIMARY OUTCOME: failed to report at least one exposure | Binary | No, reported all exposures; Yes, at least one not reported | Only nurses with an exposure; others read "Not applicable" |
| DV_PS_mean | Derived | Psychological safety mean (Q35, items a/c/e reverse-scored) | Continuous | 1.000 to 5.000 | All 872 respondents |
| DV_SC_mean | Derived | Safety climate mean (Q36) | Continuous | 1.000 to 5.000 | All 872 respondents |
| DV_POS_mean | Derived | Perceived organizational support mean (Q37) | Continuous | 1.000 to 5.000 | All 872 respondents |
