# Supplementary material for: Underreporting of blood and body fluid exposures among nurses: prevalence, reporting cascade, and modifiable organizational correlates
Source: Front Public Health. 2026 Oct 7;14:1989101. doi: 10.3389/fpubh.2026.1989101 (PMC13683579; doi:10.3389/fpubh.2026.1989101)
Supplement: Supplementary file 2 [file Table_2.DOCX]

**Table S2. Scale psychometrics: reliability, confirmatory factor analysis, exploratory factor analysis, measurement invariance, and convergent validity**

**Part A. Scale-level reliability and CFA fit**

| **Scale** | **Items** | **N** | **Cronbach's α** | **McDonald's ω** | **CFA fit** |
| --- | --- | --- | --- | --- | --- |
| Psychological Safety (Q35) | 7 | 872 | 0.821 | 0.821 | CFI=0.997, TLI=0.996, RMSEA=0.018 (0.000–0.040), SRMR=0.016 |
| Safety Climate (Q36) | 6 | 872 | 0.784 | 0.785 | CFI=0.995, TLI=0.992, RMSEA=0.027 (0.000–0.051), SRMR=0.017 |
| Perceived Org. Support (Q37) | 4 | 872 | 0.769 | 0.769 | CFI=1.000, TLI=1.006, RMSEA=0.000 (0.000–0.033), SRMR=0.003 |
| Reasons for Not Reporting (Q32) | 12 | 232 | 0.649 | 0.908 | CFI=0.853, TLI=0.793, RMSEA=0.144 (0.128–0.161), SRMR=0.143 |

**Part B. Q32 Reasons scale: item-level analysis and factor loadings**

| **Item** | **Domain** | **Description** | **Item-total r** | **CFA loading** | **EFA loading** | **EFA factor** |
| --- | --- | --- | --- | --- | --- | --- |
| Q32a | Risk minimization | Judged the risk negligible | 0.124 | 0.837 | 0.884 | F5 |
| Q32b | Risk minimization | Source patient believed uninfected | 0.130 | 0.869 | 0.823 | F5 |
| Q32c | Time/workload | Too busy to leave clinical duties | 0.214 | 0.862 | 0.815 | F4 |
| Q32d | Time/workload | Reporting process too long or complicated | 0.088 | 0.893 | 0.950 | F4 |
| Q32e | Knowledge gap | Did not know it should be reported | 0.166 | 0.921 | 0.955 | F2 |
| Q32f | Knowledge gap | Did not know how or where to report | 0.117 | 0.868 | 0.839 | F2 |
| Q32g | Fear of consequences | Feared being blamed or judged careless | 0.562 | 0.895 | 0.904 | F1 |
| Q32h | Fear of consequences | Feared effect on appraisal, contract, or promotion | 0.516 | 0.890 | 0.869 | F1 |
| Q32i | Fear of consequences | Worried colleagues or managers would find out | 0.500 | 0.855 | 0.868 | F1 |
| Q32j | Futility/embarrassment | Believed reporting would change nothing | 0.264 | 0.903 | 0.783 | F3 |
| Q32k | Futility/embarrassment | Previous report met an unhelpful response | 0.286 | 0.866 | 1.000 | F3 |
| Q32l | Futility/embarrassment | Embarrassed to admit the mistake | 0.546 | 0.085 | 0.824 | F1 |

**Part C. Measurement invariance of the Psychological Safety scale across employment status**

| **Model** | **CFI** | **TLI** | **RMSEA** | **SRMR** | **ΔCFI** |
| --- | --- | --- | --- | --- | --- |
| Configural | 1.000 | 1.002 | 0.000 | 0.017 |  |
| Metric (loadings equal) | 1.000 | 1.005 | 0.000 | 0.020 | 0.000 |

**Part D. Convergent validity: correlations among scale scores and with underreporting**

| **Variable** | **PS** | **SC** | **POS** |
| --- | --- | --- | --- |
| Psychological safety | — |  |  |
| Safety climate | 0.411 | — |  |
| Perceived org. support | 0.436 | 0.438 | — |
| Underreporting (point-biserial) | -0.267 | -0.338 | -0.138 |

Note: Part A: Cronbach's α and McDonald's ω computed on all items. CFA fit indices: CFI = comparative fit index, TLI = Tucker-Lewis index, RMSEA = root mean square error of approximation (90% CI), SRMR = standardized root mean square residual. For Q35, items a, c, e were reverse-scored before analysis. Part B: CFA is the primary analysis testing the pre-specified 5-factor structure. EFA (promax rotation, 5 factors) is an exploratory check only and cannot confirm the CFA. Item-total r = corrected item-total correlation. EFA factor = factor on which the item had its highest loading. Independent-sample confirmation of the factor structure is required. Part C: Measurement invariance tested across permanent (n = 431) vs non-permanent (n = 441) employment. ΔCFI ≤ 0.01 indicates metric invariance is supported. Part D: Pearson correlations among scale scores (range 1–5); point-biserial correlation with underreporting (0 = reported all, 1 = underreported at least one). All correlations significant at P < 0.05 unless otherwise noted. PS = psychological safety; SC = safety climate; POS = perceived organizational support.
