# Supplementary material for: Underreporting of blood and body fluid exposures among nurses: prevalence, reporting cascade, and modifiable organizational correlates
Source: Front Public Health. 2026 Oct 7;14:1989101. doi: 10.3389/fpubh.2026.1989101 (PMC13683579; doi:10.3389/fpubh.2026.1989101)
Supplement: Supplementary file 3 [file Table_3.DOCX]

**Table S3. Sensitivity analyses for factors associated with underreporting among 392 exposed nurses: negative-binomial model with exposure offset, hurdle model, and Firth penalized logistic regression**

| **Variable** | **NB IRR (95% CI)** | **NB P** | **Hurdle binary component OR (95% CI)** | **Hurdle P** | **Hurdle count component IRR (95% CI)** | **Count P** | **Firth aOR (95% CI)** | **Firth P** |
| --- | --- | --- | --- | --- | --- | --- | --- | --- |
| Age (per 5 years) | 0.97 (0.85–1.09) | 0.595 | 0.91 (0.70–1.17) | 0.458 | 1.05 (0.88–1.26) | 0.581 | 0.91 (0.71–1.17) | 0.471 |
| Rank: Charge/Supervisor vs Junior/Senior | 0.93 (0.67–1.30) | 0.691 | 0.90 (0.47–1.69) | 0.735 | 0.69 (0.38–1.24) | 0.214 | 0.89 (0.48–1.68) | 0.726 |
| Department: Moderate vs High-exposure | 0.92 (0.73–1.15) | 0.454 | 0.96 (0.56–1.63) | 0.871 | 0.82 (0.57–1.17) | 0.264 | 0.96 (0.57–1.63) | 0.878 |
| Department: Lower vs High-exposure | 1.03 (0.73–1.41) | 0.870 | 1.29 (0.61–2.76) | 0.504 | 0.89 (0.52–1.52) | 0.668 | 1.28 (0.61–2.71) | 0.518 |
| Employment: Non-permanent vs Permanent | 1.45 (1.12–1.88) | 0.005 | 2.16 (1.27–3.66) | 0.005 | 1.48 (0.98–2.26) | 0.065 | 2.11 (1.25–3.56) | 0.005 |
| Night shifts (ordinal, per level) | 1.16 (1.02–1.32) | 0.020 | 1.47 (1.09–1.98) | 0.011 | 1.16 (0.95–1.42) | 0.148 | 1.45 (1.09–1.95) | 0.011 |
| Training: None vs Any | 1.10 (0.82–1.44) | 0.525 | 1.29 (0.61–2.76) | 0.507 | 0.98 (0.63–1.52) | 0.916 | 1.27 (0.61–2.73) | 0.519 |
| Knowledge: Vague/None vs Full/General | 1.53 (1.21–1.93) | <0.001 | 3.81 (2.17–6.67) | <0.001 | 1.34 (0.94–1.92) | 0.110 | 3.64 (2.12–6.38) | <0.001 |
| Safety climate (per 1 SD) | 0.76 (0.68–0.86) | <0.001 | 0.42 (0.32–0.56) | <0.001 | 0.80 (0.66–0.96) | 0.016 | 0.44 (0.33–0.57) | <0.001 |
| Organizational support (per 1 SD) | 0.98 (0.87–1.09) | 0.676 | 1.01 (0.78–1.31) | 0.924 | 0.97 (0.82–1.14) | 0.681 | 1.01 (0.79–1.31) | 0.929 |

Note: All models use the same 10-df covariate set as Table 4 Model 1 (without psychological safety). NB = negative-binomial regression with offset log (total exposures), relating unreported exposures to total exposures; because unreported counts are bounded by total exposures, this model is supportive rather than definitive; IRR = incidence rate ratio. Hurdle model: the binary component (binomial distribution, logit link) models the odds of any unreported exposure, the same outcome as Table 4, and yields odds ratios (ORs); the count component (zero-truncated negative binomial, log link) models the number of unreported exposures among under-reporters and yields incidence rate ratios (IRRs). Firth logistic: penalized logistic regression for the binary outcome (failure to report any exposure); aOR = adjusted odds ratio. At the observed outcome prevalence (59.2%), the hurdle ORs and Firth aORs lie further from 1 than the Table 4 prevalence ratios and should not be compared numerically with them. Night-shift linearity check: likelihood ratio test comparing ordinal (1 df) vs categorical (3 df) coding: χ² = 1.34, df = 2, P = 0.511. Department sparsity check: minimum cell count = 26 (threshold = 15); 3-level grouping retained. CI = confidence interval; IRR = incidence rate ratio; OR = odds ratio; aOR = adjusted odds ratio; SD = standard deviation.
